# C1orf64 is a novel androgen receptor target gene and coregulator that interacts with 14-3-3 protein in breast cancer

**DOI:** 10.18632/oncotarget.17826

**Published:** 2017-05-11

**Authors:** Ali Naderi

**Affiliations:** ^1^ University of Hawaii Cancer Center, Cancer Biology Program, Honolulu, Hawaii 96813, USA

**Keywords:** androgen receptor, C1orf64, 14-3-3, breast cancer, coregulator

## Abstract

This study investigated the network of genes that are co-expressed with androgen receptor (AR) to discover novel AR targets in breast cancer. Bioinformatics analysis of two datasets from breast cancer cell lines resulted in the identification of an AR-gene signature constituted of 98 genes that highly correlated with AR expression. Notably, C1orf64 showed the highest positive correlation with AR across the datasets with a correlation coefficient (CC) of 0.737. In addition, C1orf64 closely correlated with AR expression in primary and metastatic breast tumors and C1orf64 expression was relatively higher in breast tumors with a lower grade and lobular histology. Furthermore, there is a functional interplay between AR and C1orf64 in breast cancer. In this process, AR activation directly represses C1orf64 transcription and C1orf64, in turn, interacts with AR as a corepressor and negatively regulates the AR-mediated induction of prolactin-induced protein (PIP) and AR reporter activity. Moreover, the corepressor effect of C1orf64 results in a reduction of AR binding to PIP promoter. The other aspect of this interplay involves a cross-talk between AR and estrogen receptor (ER) signaling in which C1orf64 silencing intensifies the AR-mediated down-regulation of ER target gene, progesterone receptor. Therefore, the repression of C1orf64 by AR provides an underlying mechanism for the AR inhibitory effects on ER signaling. To elucidate the biochemical mechanisms of C1orf64 function, this study demonstrates that C1orf64 is a phosphothreonine protein that interacts with the chaperone protein 14-3-3. In summary, C1orf64 is a novel AR coregulator and a 14-3-3 binding partner in breast cancer.

## INTRODUCTION

Recent advancements in the molecular profiling and cancer genomics have provided the opportunity for a better understanding of breast cancer biology and the discovery of novel therapeutic targets in this disease. In this process, androgen receptor (AR) has been identified as a key molecular marker in breast cancer that is expressed in 90% of estrogen receptor (ER)-positive and 50% of ER-negative breast tumors [1–4]. The subtype of ER-negative breast cancer that has a high level of AR expression is termed “molecular apocrine” and is characterized by a steroid-response gene signature that includes AR, FOXA1, and prolactin-induced protein (PIP), [5–7]. These findings have resulted in a growing interest in the understanding of molecular functions and therapeutic implications of AR in breast cancer.

Studies conducted by my group and others have revealed that AR as a transcription factor has an important function in the regulation of key signaling pathways in breast cancer [4, 8–12]. This includes a positive feedback loop between AR and ErbB2-extracellular signal-regulated kinase (ERK) signaling in molecular apocrine cells [4, 9, 12]. In this feedback loop, AR regulates ERK phosphorylation through the transcriptional activation of ErbB2 and, in turn, the ERK-CREB1 signaling regulates AR transcription [9]. Furthermore, AR acts as a transcriptional activator of PIP, a characteristic biomarker in breast cancer, which is required for cell cycle progression in both ER-positive and ER-negative breast cancer cells [8, 11, 13–15]. Moreover, studies on molecular apocrine cell line MDA-MB-453 demonstrated that AR induces the WNT7B-ErbB3 signaling and there is a transcriptional interaction between AR and FOXA1 in these cells [10, 16]. Importantly, multiple preclinical studies have suggested a potential role for AR as a therapeutic target in breast cancer and currently this topic is under active investigation in clinical trials [4, 6, 12, 17–19]. Despite the emerging data on the importance of AR function in breast cancer, the available studies have been mostly conducted on a limited number of cell line models and broader molecular functions of AR in breast cancer including key targets and coregulators of this gene have remained largely unknown.

To address these shortcomings, my group has recently examined gene expression data from a cohort of 52 breast cancer cell lines to identify a network of AR co-expressed genes [20]. This study has identified an “AR-gene signature” composed of 35 genes that were highly co-expressed with AR across the dataset (absolute correlation coefficient |CC|> 0.6), [20]. In this process, gene encoding factor VII (F7) was identified as a novel AR target gene. Importantly, AR activation in breast cancer cells induces endogenous factor VII (FVII) activity to convert factor X to Xa in conjunction with the tissue factor (TF), [20]. This activation of coagulation FVII by AR provides a novel mechanism for the transcriptional regulation of ectopic FVII expression in cancer cells. In addition, this model implicates a potential role for AR signaling in the pathobiology of thromboembolic events and regulation of FVII/TF signaling in breast cancer [20].

Therefore, the study of AR co-expressed genes in large genomic datasets would provide an innovative approach to investigate AR molecular functions and novel target genes in breast cancer. This approach ensures that different molecular subtypes of breast cancer are represented in the study and the identified genes are highly correlated with AR expression in a large non-biased model of this disease. Importantly, the identified AR co-expressed genes are likely to include biologically significant AR target genes and transcriptional coregulators that are present across different subtypes of breast cancer. In the current study, this approach has been further explored by a combined analysis of two large expression datasets in breast cancer to identify an AR-gene signature of highly co-expressed genes followed by the transcriptional and functional studies of some of the key findings. Notably, this study identifies a poorly-understood gene “C1orf64” as both a novel target gene and a coregulator of AR in breast cancer that interacts with 14-3-3 protein.

## RESULTS

### A transcriptional signature of AR co-expressed genes in two datasets

To identify a gene-signature that highly correlates with AR expression in breast cancer, two expression microarray datasets were analyzed from studies published by Neve *et al*. (study 1) and Kao *et al*. (study 2) as explained in methods [21, 22]. It is notable that study 1 and study 2 included 52 and 50 breast cancer cell lines, respectively. In addition, a total of 37 cell lines were in common and a total of 28 cell lines varied between the two cohorts. Initially, a list of highly co-expressed genes with AR was identified in each dataset using a cutoff of |CC| of ≥ 0.6, p< 0.001 and then a combined “AR-gene signature” was generated by compiling the subset of AR co-expressed genes in both cohorts (Table 1 and Supplementary Table 1). Furthermore, genes represented on both microarray platforms that had a significant correlation with AR expression in each of the datasets (p< 0.05) were identified (Table 1 and Supplementary Table 1).

**Table 1:** A combined list of highly co-expressed genes with AR in two datasets

Gene (POS)	CC	Gene (POS)	CC	Gene (NEG)	CC	Gene (NEG)	CC
*C1orf64*	0.737	*EMP2	0.630	*PRNP	−0.750	*AKR1B1	−0.614
*SIDT1*	0.730	*CREB3L4*	0.629	*LAMA2*	−0.686	*GLS	−0.613
*F7	0.716	*SLC16A6	0.628	*DONSON*	−0.681	*STIL*	−0.612
*PATZ1*	0.709	*KIAA1324	0.627	*LARP6*	−0.679	*TOP2A*	−0.612
*ZNF205-AS1*	0.699	*GGCT*	0.623	*NFIL3	−0.674	*LINC00597	−0.612
*RHOH	0.693	*DEGS2*	0.622	*APOBEC3C*	−0.673	*BUB1	−0.609
*CCDC125*	0.689	*PCDHA5*	0.621	*ETS1*	−0.672	*GART*	−0.609
*NFATC4*	0.681	*TRIL*	0.620	*NAB1	−0.671	*ANXA1	−0.609
*LRFN2*	0.678	*DALRD3*	0.616	*IGF2BP2*	−0.664	*CENTD3*	−0.609
*TBC1D30*	0.673	*REEP6*	0.615	*ECM2*	−0.663	*WIPF1*	−0.609
*TFAP2B	0.673	*SLC9A1*	0.614	*TTK*	−0.662	*NXN*	−0.604
*SPDEF	0.670	*UMODL1*	0.614	*KIRREL*	−0.661	*CMTM7*	−0.603
*BCAS1	0.662	*IGHM*	0.612	*S100A3	−0.655		
*MXD4*	0.661	*HPX	0.612	*EHBP1	−0.654		
*IRX5	0.659	*GATA3	0.610	*INPP1	−0.652		
*CACNA1D	0.656	*PLEKHF2*	0.610	*PICALM*	−0.649		
*DOPEY2*	0.656	*TFF3	0.607	*USP1*	−0.642		
*RHOB	0.654	*GSE1*	0.605	*ANKS6*	−0.641		
*CTNND2	0.651	*RND1*	0.605	*ncRNA miR-221*	−0.639		
*SLCO2A1*	0.645	*AMBP*	0.603	*CAV2	−0.637		
*FOXA1	0.644	*ZG16B*	0.603	*TUBB6*	−0.634		
*FOXR1*	0.644	*TPRN*	0.602	*FYN	−0.633		
*SGSM3*	0.638	*CRAT	0.601	*GALNT2	−0.630		
*SLC9A2*	0.637	*MVK*	0.601	*PGM1	−0.628		
*TP53TG1*	0.636	*CACFD1	0.601	*POPDC3*	−0.624		
*FGFR4	0.634	*SLC9A3R1	0.601	*CAPG*	−0.622		
*PYGO1*	0.633	*ATP8A1*	0.600	*CAV1	−0.622		
*MGAT5*	0.632	*HMGCS2	0.600	*FHL3*	−0.617		
*TTC6*	0.631			*PIM1	−0.615		

All genes have lCCl values ≥ 0.6 with AR expression at a p< 0.001 in at least one dataset. Asterisks indicate genes that are present in both datasets and have a significant CC (p< 0.05) with AR. The strongest CC values for these genes are presented. CC: Pearson's correlation coefficient. (POS) and (NEG) indicate positive and negative CC values, respectively.

The combined AR-gene signature included a total of 98 genes apart from AR itself (Table 1 and Supplementary Table 1). Importantly, C1orf64 showed the highest positive correlation with AR expression in this signature with a CC value of 0.737 (Table 1). Furthermore, there were only three other genes in the AR-signature that had a positive CC > 0.7 namely, SIDT1, F7, and PATZ1 (Table 1). It is notable that F7, which we have previously identified as an AR target gene [20], demonstrated a significant correlation with AR expression in both datasets (Table 1 and Supplementary Table 1). In addition, a total of 52 genes in the combined AR-signature overlapped between the two expression microarray platforms and among these a subset of 38 genes showed significant CC values with AR expression (p< 0.05) in both cohorts (Table 1 and Supplementary Table 1).

Next, functional annotations of the combined AR-gene signature were assessed using DAVID Bioinformatics Resources (Table 2). In this process, fold enrichment (FE) and p value for each functional term in GOTERM and INTERPRO categories were obtained (Table 2). Notably, there were five significant GOTERM functional terms in the AR-signature (p< 0.05) and the top two categories included “sequence-specific DNA binding” (FE: 3, p= 0.005) and “transcription factor binding” (FE: 2.9, p= 0.02), (Table 2). Furthermore, INTERPRO analysis of the signature predicted four significant functional terms (p< 0.05) and the top two categories were identified as “Ras small GTPase” (FE: 30, p= 0.004) and “Winged helix repressor DNA-binding” (FE: 5.4, p= 0.013), (Table 2). Therefore, the combined analysis of two large expression microarray datasets reveals an AR- gene signature that is significantly enriched for genes involved in DNA binding and transcriptional activities. Importantly, C1orf64 is identified as the gene with the closest correlation with AR expression in the AR-gene signature.

**Table 2:** Functional annotation of combined AR signature genes in two datasets

Category	Term	PValue	Genes	FE
GOTERM	sequence-specific DNA binding	0.005	*AR, IRX5, ETS1, GATA3, FOXA1, SPDEF, TFAP2B, CREB3L4, NFIL3, FOXR1*	3
GOTERM	transcription factor binding	0.020	*ETS1, GATA3, PIM1, TFAP2B, NFATC4, NFIL3, SLC9A1, MXD4*	2.9
GOTERM	protein dimerization activity	0.026	*AMBP, CAV2, AR, GGCT, TFAP2B, CREB3L4, NFIL3, TOP2A*	2.7
GOTERM	sodium:hydrogen antiporter activity	0.047	*SLC9A2, SLC9A1*	41
GOTERM	nitric-oxide synthase binding	0.047	*CAV2, CAV1*	41
INTERPRO	Ras small GTPase, Rho type	0.004	*RND1, RHOB, RHOH*	30
INTERPRO	Winged helix repressor DNA-binding	0.013	*LARP6, ETS1, FOXA1, SPDEF, FOXR1*	5.4
INTERPRO	Caveolin	0.014	*CAV2, CAV1*	134
INTERPRO	Na+/H+ exchanger, conserved region	0.043	*SLC9A2, SLC9A1*	45

Gene Ontology (GO) and protein annotations are shown for the signature using DAVID Bioinformatics Resources. Functional annotation are analyzed at a significance level of p< 0.05. FE: Fold Enrichment.

### AR regulates the transcription of its co-expressed genes

In view of a strong correlation between the expression of AR and each of the signature genes, it is likely that some of these genes are regulated by AR activation in breast cancer cells. In this respect, a previous study by my group has demonstrated that F7 (CC: 0.716) is a direct AR target gene [20]. Therefore, to identify novel AR targets, the effect of AR activation was assessed on two genes that showed the highest positive correlation with AR expression in the signature namely, C1orf64 and SIDT1 (Table 1). In addition, the effect of AR activation was also assessed on the top three genes that significantly correlated with AR expression in both datasets namely, RHOH, TFAP2B, and SPDEF (Table 1). The transcriptional studies were carried out using breast cancer cell lines T-47D (ER+/AR+, luminal A subtype) and MFM-223 (ER-/AR+, molecular apocrine subtype), [14, 20]. AR activation was carried out using dihydrotestosterone (DHT) treatment at 10 nM concentration at three time-points of 6h, 24h, and 48h as explained in methods. Next, the effect of DHT on fold changes in gene expression was measured using quantitative real time-polymerase chain reaction (qRT-PCR) relative to controls and all experiments were carried out in six replicates.

**Figure 1:**
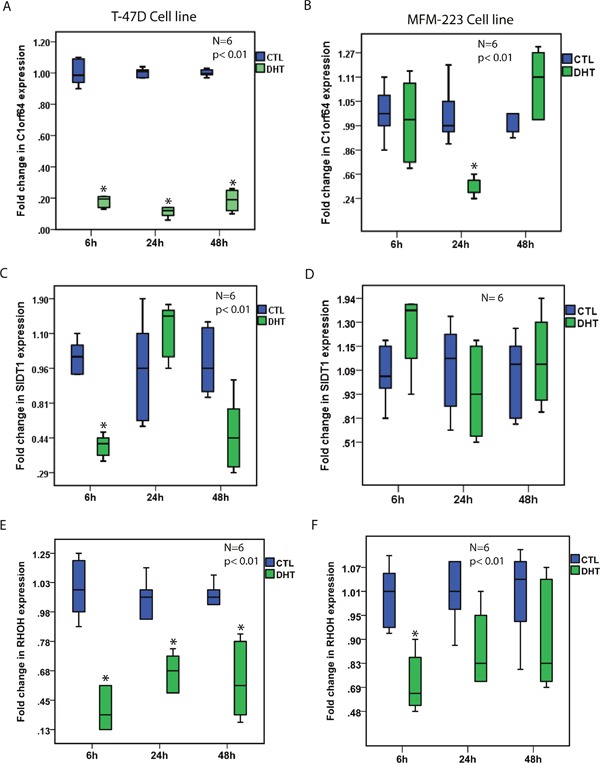
The effect of AR activation on the expression of AR-signature genes **(A and B)** Fold changes in C1orf64 expression using qRT-PCR after DHT treatment at 10 nM in T-47D and MFM-223 cell lines at 6h, 24h, and 48h time-points. Fold changes are relative to the control (CTL) experiments at each time-point. *, p< 0.01 is for DHT-treated vs. control groups. **(C and D)** Fold changes in SIDT1 expression using qRT-PCR after DHT treatment at 10 nM in T-47D and MFM-223 cell lines at 6h, 24h, and 48h time-points. **(E and F)** Fold changes in RHOH expression using qRT-PCR after DHT treatment at 10 nM in T-47D and MFM-223 cell lines at 6h, 24h, and 48h time-points. *, p< 0.01 is for DHT-treated vs. control groups.

**Figure 2:**
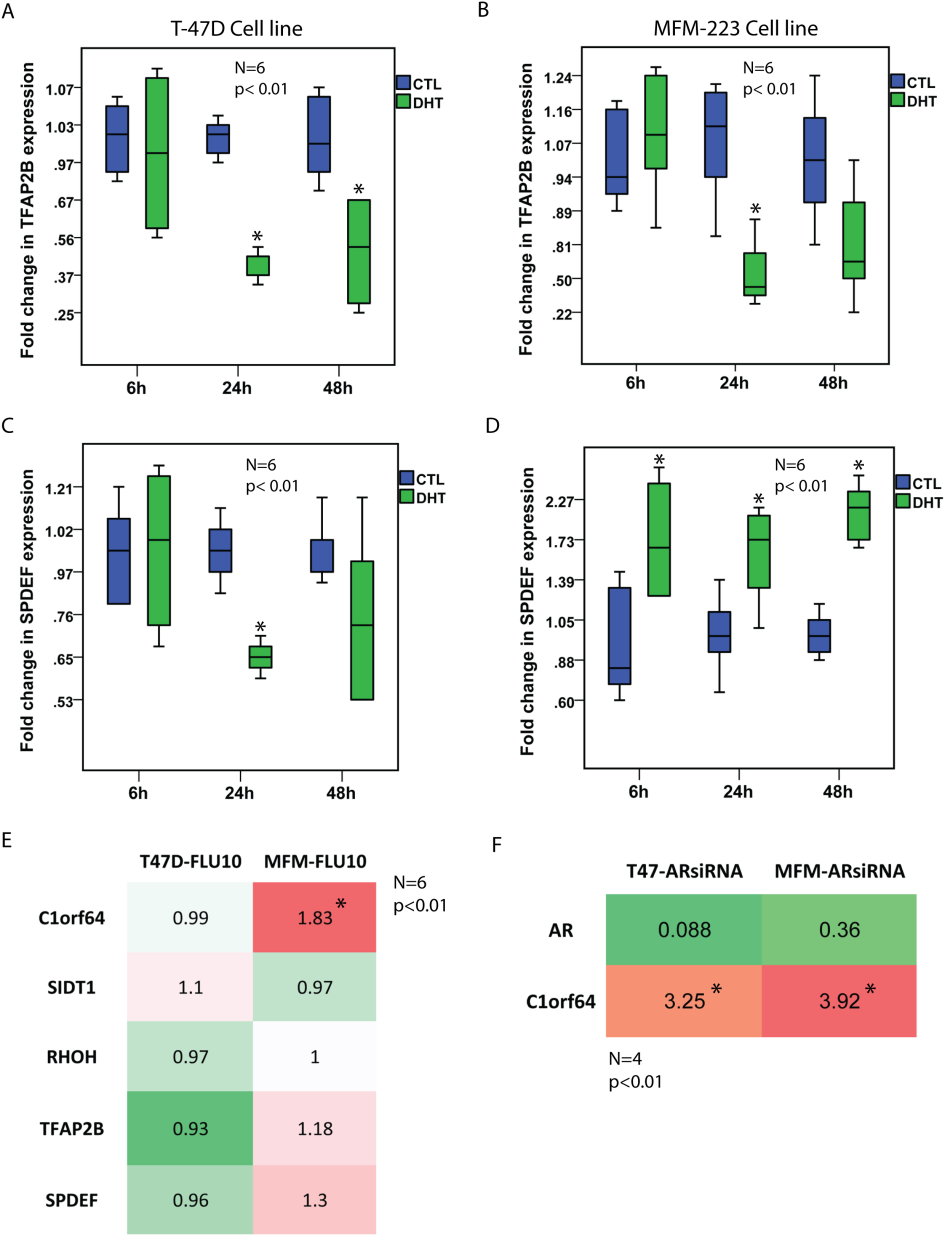
The effects of AR activation and inhibition on the expression of AR-signature genes **(A and B)** Fold changes in TFAP2B expression using qRT-PCR after DHT treatment at 10 nM in T-47D and MFM-223 cell lines at 6h, 24h, and 48h time-points. Fold changes are relative to the control (CTL) experiments at each time-point. *, p< 0.01 is for DHT-treated vs. control groups. **(C and D)** Fold changes in SPDEF expression using qRT-PCR after DHT treatment at 10 nM in T-47D and MFM-223 cell lines at 6h, 24h, and 48h time-points. *, p< 0.01 is for DHT-treated vs. control groups. **(E)** A Heat map to show fold changes in the expression of C1orf64, SIDT1, RHOH, TFAP2B, and SPDEF genes using qRT-PCR after flutamide (FLU) treatment at 10 μM for 24h in T-47D and MFM-223 cell lines. Expression values are relative to the controls. Experiments were carried out in 6 replicates and *, p< 0.01 is for FLU-treated vs. control group. Red color denotes up-regulation and green color indicates down-regulation. **(F)** A Heat map to show fold changes in AR and C1orf64 expression following AR-silencing with an AR-siRNA duplex in T-47D and MFM-223 cell lines. Fold changes are measured using qRT-PCR. Experiments were carried out in 4 replicates and *, p< 0.01 is for C1orf64 expression in AR-siRNA vs. control-siRNA.

Notably, DHT treatment resulted in a marked reduction of C1orf64 expression in T-47D cells at all three time-points by 5 to 10-fold and also led to a 2-fold reduction of C1orf64 expression in MFM-223 cells at the 24h time-point (p< 0.01, Figure 1A and 1B). Furthermore, DHT treatment decreased SIDT1 expression by 2.5-fold in T-47D cells after 6h (p< 0.01, Figure 1C); however, it did not significantly change the level of this gene in MFM-223 cells (Figure 1D). In addition, there was a significant reduction of RHOH expression following DHT treatment in T-47D cells at all three time-points by 1.7 to 3.3-fold (p< 0.01, Figure 1E) as well as a 1.7-fold decrease in the expression of this gene in MFM-223 cells after 6h (p< 0.01, Figure 1F). Moreover, TFAP2B expression was significantly reduced by approximately 2.2-fold at 24h and 48h time-points in T-47D cells and by 1.75-fold in MFM-223 cells at 24h (p< 0.01, Figure 2A and 2B). Finally, although there was a modest reduction of SPDEF expression following DHT treatment in T-47D cells at 24h time-point by 1.6-fold, the expression of this gene was consistently increased following DHT treatment in MFM-223 cells at all three time-points by 1.6 to 2.3-fold (p< 0.01, Figure 2C and 2D).

Next, to assess the effect of AR inhibition on the expression levels of C1orf64, SIDT1, RHOH, TFAP2B, and SPDEF genes, T-47D and MFM-223 cell lines were treated with AR antagonist flutamide at 10 μM concentration for 24h and fold changes in gene expression were then measured using qRT-PCR relative to the control experiments. It is notable that AR inhibition led to a significant increase in C1orf64 expression by 1.83-fold in MFM-223 cell line (p< 0.01, Figure 2E). However, there was no significant change in the expression of other genes following flutamide treatment (Figure 2E). Furthermore, the effect of AR silencing using an AR-siRNA duplex (AR-siRNA) was examined on C1orf64 expression. AR silencing efficiencies were assessed using qRT-PCR. Notably, AR silencing reduced AR expression by 91% and 64% in T-47D and MFM-223 cell lines, respectively (Figure 2F). Moreover, AR silencing led to a significant increase in C1orf64 expression by 3.25 and 3.92-fold in T-47D and MFM-223 lines, respectively (p< 0.01, Figure 2F). All together, these findings suggest that AR activation regulates the transcription of a number of AR co-expressed genes and has a profound repressive effect on C1orf64 expression in breast cancer cells.

### C1orf64 and SPDEF are AR target genes

**Figure 3:**
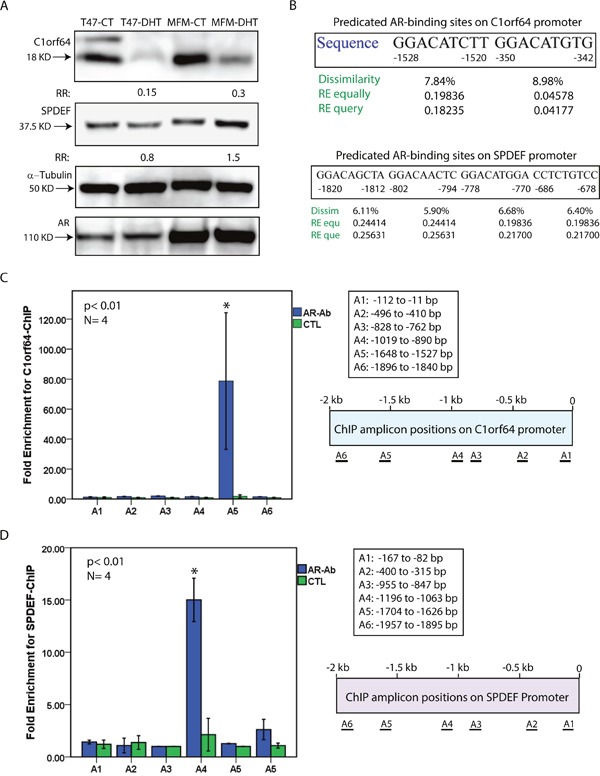
Western blot analysis and ChIP assays for C1orf64 and SPDEF **(A)** immunoblotting to assess C1orf64, SPDEF, and AR protein levels in T-47D and MFM-233 cell lines following DHT treatment at 10 nM for 24h. Fold change (RR) in each band density was measured relative to control in three replicate experiments. A mouse α-tubulin antibody was applied to assess loading. **(B)** Predicated AR-binding sites in the 2 Kb promoter region of C1orf64 and SPDEF genes using PROMO software. Binding sites were predicted within a dissimilarity margin ≤ 15%. Dissimilarity (Dissim) margins, and Random Expectation (RE) values using RE equally (equ) and RE query (que) models for the predicated sites are shown. **(C)** ChIP assays for C1orf64 promoter using AR antibody (AR-Ab) to assess AR binding to each promoter region (A1-A6). Amplification of 1% of input chromatin was used as the input control and a non-specific antibody (CTL) served as negative control. ChIP assays were carried out in four replicates and fold enrichment is shown for each amplicon. ChIP amplicon positions (A1-A6) on C1orf64 promoter are shown. *, p< 0.01 is for AR-Ab vs. CTL. Error Bars: ± 2SEM. **(D)** ChIP assays for SPDEF promoter using AR antibody to assess AR binding to each promoter region (A1-A6) as outlined in **(C).**

In view of the fact that C1orf64 was markedly repressed by AR activation and SPDEF was the only AR-induced gene in the tested subset, these two genes were selected to further examine as possible direct targets of AR in breast cancer cells. In this process, first the effect of DHT treatment at 10 nM for 24h was assessed on the protein levels of C1orf64 and SPDEF in T-47D and MFM-223 cell lines. Western blot analysis was then carried out on the extracted lysates from the DHT-treated and control samples to examine the protein levels. Western blots were performed in three replicates and fold changes in band intensity were measured following DHT treatment relative to the control samples. Importantly, DHT treatment markedly reduced C1orf64 protein levels by 6.7-fold (15% of control) and 3.3-fold (30% of control) in T-47D and MFM-223 cell lines, respectively (Figure 3A). In addition, SPDEF protein level was slightly decreased by 1.25-fold in T-47D cells and increased by 1.5-fold in MFM-223 cells following DHT treatment (Figure 3A). Furthermore, immunoblotting for AR demonstrated that there is no change in the AR protein levels following DHT treatment and the baseline expression of AR is higher in MFM-223 compared to T-47D cell line (Figure 3A). Therefore, the effect of DHT-mediated AR activation on the protein levels of C1orf64 and SPDEF is consistent with its effect on the transcription of these genes.

To examine whether C1orf64 and SPDEF are AR target genes, the 2 kb promoter regions of these genes were assessed for predicated AR-binding sites using PROMO software as explained in methods. This analysis identified two predicated sites for AR binding in -1528 to -1520 and -350 to -342 bp regions of C1orf64 promoter (Figure 3B). In addition, there were four predicated AR binding sites in -1820 to -1812, -802 to -794, -778 to -770, and -686 to -678 bp regions of SPDEF promoter (Figure 3B). Furthermore, promoter regions of C1orf64 and SPDEF were examined for open chromatin regions that are accessible to transcription. This analysis was carried out based on the data from ENCODE project for DNase I Hypersensitivity Sites (DHSs) and H3K27ac Mark using the UCSC Genome Browser as explained in methods. Notably, the 1.8 kb promoter regions of both C1orf64 and SPDEF genes contain DHSs and H3K27ac Mark in multiple cell types, which indicates the accessibility of these promoters for transcriptional activity (Supplementary Figure 1).

Next, Chromatin immunoprecipitation (ChIP) assays were carried out to investigate direct AR binding to C1orf64 and SPDEF promoters. In this process, a total of six primer sets were employed for each of the C1orf64 and SPDEF 2 kb promoter regions to generate amplicons using qRT-PCR (Figure 3C). Both T-47D and MFM-223 cell lines were applied for ChIP assays and a total of four replicate experiments were carried out for each primer set. Following qRT-PCR, fold enrichments for ChIP-signal were calculated as described in methods. ChIP assays revealed a significant enrichment by 80-fold (± 45), p< 0.01 for AR binding to the region of A5 amplicon (-1648 to -1527 bp) on C1orf64 promoter (Figure 3C). Notably, this region overlaps with one of the two predicated AR-binding sites based on the bioinformatics analysis (Figure 3B). In addition, the other regions of C1orf64 did not demonstrate a significant enrichment for AR binding (Figure 3C). Furthermore, ChIP assays revealed a significant enrichment by 15-fold (± 2), p< 0.01 for AR binding to the A4 amplicon region (-1196 to -1063 bp) on SPDEF promoter; however, there was no significant AR binding to the other regions of this promoter (Figure 3D). This identified A4 amplicon region is within 300 bp of two of the predicated AR-binding sites on SPDEF promoter (Figure 3B). Collectively, these findings suggest that C1orf64 and SPDEF are direct transcriptional targets of AR in breast cancer.

### There is a close association between the AR-signature and AR promoter binding

The AR-gene signature was examined for possible AR-binding sites using publically available AR ChIP-sequencing (ChIP-seq) data in breast cancer cells. In this process, a list of AR-signature genes with a detectable AR-binding signal in their 5 kb promoter regions were identified (Table 3). Next, an average AR ChIP-seq signal was calculated across three replicate experiments for each promoter binding detected in the signature genes. A total of 31 genes in the AR-signature, including C1orf64 and SPDEF, had detectable AR ChIP-seq signals (Table 3). In addition, F7, which was identified as an AR target gene in our previous study [20], also showed an AR binding in ChIP-seq analysis (Table 3). It is notable that 30 out of 57 positively co-expressed genes in the AR-signature (53%) had ChIP-seq detected AR binding (Table 3). In contrast, only one negatively co-expressed gene, PIM1, was among this subset. Furthermore, SITD1 and RHOH also contained a detectable signal in AR ChIP-seq (Table 3).

**Table 3:** A list of AR-signature genes that have AR-binding signals in their 5 kb promoter regions using ChIP-seq data

Gene	AR average	C1orf64 CC	p value	Datasets
*C1orf64	165	1	<0.001	BC Cell lines
*SIDT1	133	0.667	<0.001	TCGA Breast
*F7	58	0.678	<0.001	BC Cell lines
*PATZ1*	38		>0.05	TCGA Breast
*RHOH	116	0.629	<0.001	BC Cell lines
*CCDC125*	267		>0.05	TCGA Breast
*TBC1D30*	119		>0.05	Bos Breast
*SPDEF	51	0.734	<0.001	BC Cell lines
*DOPEY2	230	0.693	<0.001	BC Cell lines
*RHOB	216	0.677	<0.001	BC Cell lines
*SLCO2A1*	30		>0.05	BC Cell lines
*FOXA1	174	0.721	<0.001	BC Cell lines
*SLC9A2	60	0.809	<0.001	BC Cell lines
*PYGO1*	27		>0.05	BC Cell lines
*TTC6	174	0.631	<0.001	BC Cell lines
*CREB3L4	27	0.775	<0.001	BC Cell lines
*SLC16A6	839	0.505	0.001	BC Cell lines
*KIAA1324	46	0.727	<0.001	BC Cell lines
*DEGS2	498	0.665	<0.001	BC Cell lines
*TRIL*	45		>0.05	TCGA Breast
*DALRD3*	293	NA		
*UMODL1	42	0.675	<0.001	BC Cell lines
*GSE1*	19		>0.05	TCGA Breast
*AMBP*	333		>0.05	BC Cell lines
*ZG16B	107	0.577	<0.001	TCGA Breast
*CRAT	654	0.42	0.009	BC Cell lines
*MVK*	46	NA		
*CACFD1*	120	NA		
*SLC9A3R1	101	0.659	<0.001	BC Cell lines
*ATP8A1	34	0.664	<0.001	BC Cell lines
*PIM1	44	−0.632	<0.001	BC Cell lines

Average ChIP-seq signal for AR binding, correlation coefficient (CC) between the expression of each signature gene and C1orf64, p value for each CC, and the source of data for CC analysis are shown. BC: breast cancer, NA: not available. Asterisks depict the AR-signature genes that have a detectable AR-binding signal using ChIP-seq and a significant correlation (p<0.01) with C1orf64 expression in breast cancer.

Next, CC values were calculated between C1orf64 expression and the other 30 AR-signature genes that were identified in the AR ChIP-seq analysis. In this subset, a total of 19 genes demonstrated a significant CC value with C1orf64, including SPDEF that had a CC of 0.734 (p<0.001, Table 3). Furthermore, the co-expression pattern between the genes in this subset and C1orf64 was uniformly in the same direction as that observed with AR co-expression (Table 3). These findings further confirm that C1orf64 and SPDEF are AR target genes in breast cancer and also suggest that a positive coexpression with AR can be applied as a predictor of AR target genes in combination with a detectable binding in AR ChIP-seq. Moreover, the close correlation between C1orf64 expression and 63% (19 out of 30) of the AR-signature genes, which contain a ChIP-seq signal, indicates that C1orf64 is co-expressed with a large number of AR target genes.

### C1orf64 is co-expressed with AR in breast tumors

**Figure 4:**
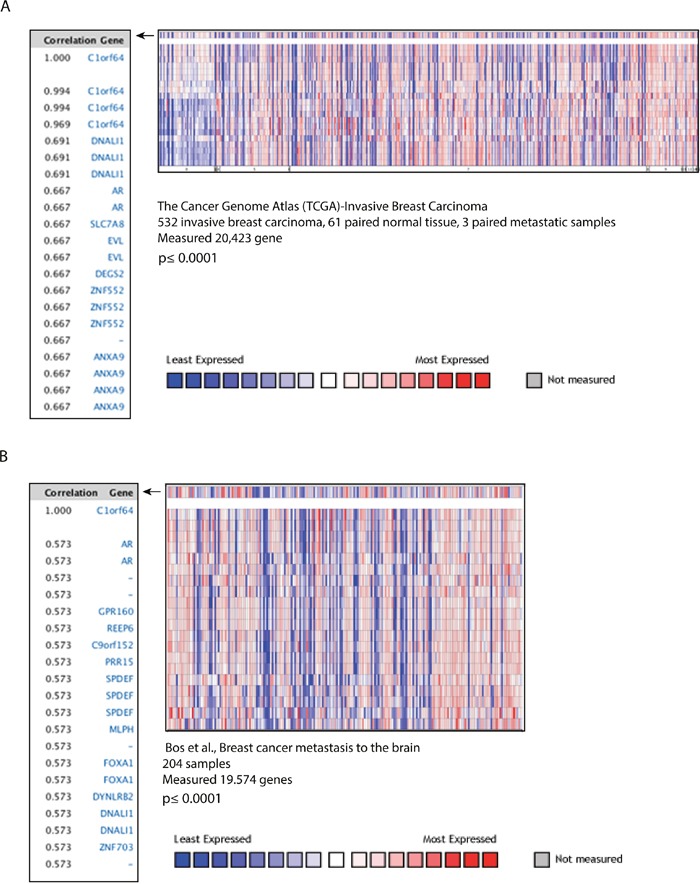
C1orf64 co-expressed genes in breast tumors **(A)** Heat map and correlation coefficients of C1orf64 co-expressed genes in TCGA-Invasive Breast Carcinoma Gene Expression Data analyzed using *ONCOMINE*. Correlation values are shown for the top co-expressed genes with C1orf64 (p≤ 0.0001). Heat map depicts the gene expression pattern across the tumor samples. **(B)** Heat map and correlation coefficients of C1orf64 co-expressed genes analyzed using *ONCOMINE* in a dataset published by Bos *et al*. Correlation values of the top co-expressed genes with C1orf64 (p≤ 0.0001) and a heat map for gene expression across the cohort are shown.

To further investigate the association between C1orf64 and AR in breast cancer, C1orf64 co-expressed genes were assessed in primary and metastatic breast tumors. In this respect, two large studies that contained both primary and metastatic breast tumor samples were analyzed using *ONCOMINE* database [23–25]. The first dataset constituted of TCGA-Invasive Breast Carcinoma Gene Expression Data with a total of 532 invasive breast carcinoma, 61 paired normal breast tissue, and 3 paired metastatic samples [24]. Co-expression analysis in this dataset was carried out to identify genes with the highest correlations with C1orf64 expression (Figure 4A). Importantly, AR showed one of the top two correlations with C1orf64 expression in this cohort with a CC value of 0.667 (p≤ 0.0001, Figure 4A). The second dataset was obtained from a study conducted by Bos *et al*. and contained a total of 204 breast carcinoma samples with brain metastasis [25]. Notably, co-expression analysis of this cohort for C1orf64 also revealed that AR has one of the highest correlations with C1orf64 expression across the dataset with a CC value of 0.573 (p≤ 0.0001, Figure 4B). Furthermore, SPDEF showed a strong correlation with C1orf64 expression in this dataset with a CC of 0.573 (p≤ 0.0001, Figure 4B). These findings further prove that C1orf64 and AR are closely co-expressed across various subtypes and clinical stages of breast cancer with reproducible findings using different cohorts.

### C1orf64 is associated with a lower grade and lobular breast tumors

The association of C1orf64 expression with clinical and pathological features of breast tumors was investigated using datasets available in *ONCOMINE* as described in methods. In this respect, C1orf64 log2 median expression values were analyzed to assess a differential expression of C1orf64 for histology type, tumor grade, ER status, ErbB2 status, triple negative (TN) status, and outcome across twenty-two breast cancer datasets. Notably, there was a significantly higher expression of C1orf64 in grade 1 and 2 tumors compared to grade 3 cases by 1.5 and 1.3-fold, respectively (p< 0.02, Table 4). In addition, C1orf64 expression was 2.6-fold higher in lobular histology compared to ductal (p= 0.02, Table 4), and it was also relatively higher in ER-positive and non-TN tumors by 2.6 and 1.8-fold, respectively (p< 0.01, Table 4). However, there was no significant association between C1orf64 expression and ErbB2 status or outcome in breast tumors (Table 4). These results suggest that C1orf64 is differentially expressed in some of the clinical and pathological subtypes of breast cancer.

**Table 4:** Association of C1orf64 expression with clinical and pathological features in breast cancer

Feature	Histology		Grade			ER		ErbB2		TN status		Outcome	
Subtype	ductal	lobular	1	2	3	Neg	Pos	Neg	Pos	TN	others	good	poor
C1orf64 log2	0.58	1.96	1.07	0.82	0.46	0.03	1.42	0.52	0.61	0.02	0.87	1.44	1.27
p value	0.02*		<0.02*			<0.01*		>0.05		<0.01*		>0.05	
Fold difference		2.6	1.5	1.3			2.6	N/A			1.8	N/A	
Number of datasets	15			18		18		17		16		8	

C1orf64 differential analysis was carried out using the breast cancer datasets available in *ONCOMINE*.

P values are for the following comparisons: ductal vs. lobular; grade 1 vs. grade 3; grade 2 vs. grade 3; ER-negative (Neg) vs. ER-positive (Pos); ErbB2-neg vs. ErbB2-pos; triple negative (TN) vs. other biomarker status; good outcome (good: no recurrence or death in 5 years) vs. poor (recurrence or death in 5 years). C1orf64 expression log2 values are the median of measurements across the datasets. The number of datasets analyzed for each subtype is shown and fold difference in C1orf64 expression is presented for each significant p value. P values are calculated using Wilcoxon Signed Rank Test. *, depicts significance at < 0.05. N/A: not applicable.

### C1orf64 represses the AR-mediated induction of PIP

The fact that AR is widely co-expressed with C1orf64 in breast cancer and negatively regulates the expression of this gene, raises the question of a possible biological significance for such a marked repression of C1orf64 by AR in breast cancer cells. In this respect, a plausible hypothesis is that C1orf64, in turn, may have a negative regulatory effect on AR function in breast cancer, which would provide a biological advantage for AR to repress C1orf64 expression in order to sustain its own transcriptional activity. To investigate this hypothesis, the effect of C1orf64 expression on the AR-mediated transcriptional activation of PIP was examined in breast cancer cell lines T-47D and MFM-223. It is notable that PIP is an established transcriptional target of AR and AR activation is necessary and sufficient for PIP expression in breast cancer cells [8, 11, 14, 15, 26, 27]. Therefore, the study of C1orf64 effect on PIP expression provides a valid model to examine a possible regulatory effect for C1orf64 on AR transcriptional activity in breast cancer.

**Figure 5:**
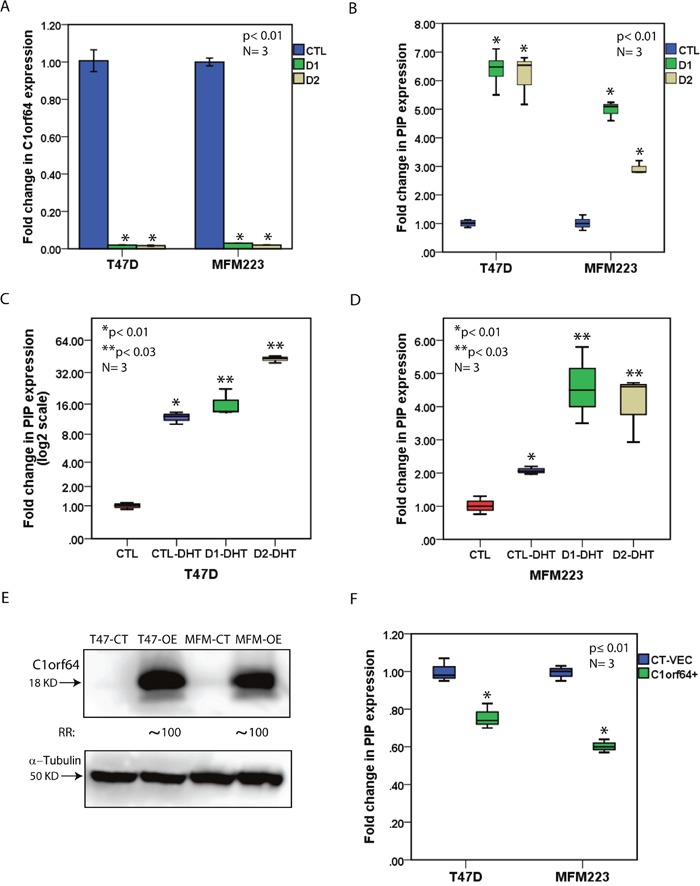
The effect of C1orf64 on AR-mediated induction of PIP **(A)** qRT-PCR to demonstrate C1orf64 silencing efficiencies with siRNA-D1 (D1) and siRNA-D2 (D2) in T-47D and MFM-223 cell lines. C1orf64 expression is relative to non-targeting siRNA (CTL). *, p< 0.01 is for D1 or D2 vs. CTL. Error Bars: ± 2SEM. **(B)** qRT-PCR to show the effect of C1orf64 silencing using siRNA-D1 (D1) and siRNA-D2 (D2) on PIP expression in T-47D and MFM-223 cells. *, p< 0.01 is for D1 or D2 vs. CTL. **(C)** qRT-PCR to assess the effect of C1orf64 silencing on the DHT-mediated induction of PIP expression in T-47D cells. CTL: CTL-siRNA + solvent control, CTL-DHT: CTL-siRNA + DHT, D1-DHT: siRNA-D1 + DHT, D2-DHT: siRNA-D2 + DHT. *, p< 0.01 is for CTL vs. CTL-DHT and **, p< 0.03 is for CTL-DHT vs. D1-DHT or D2-DHT. **(D)** qRT-PCR to examine the effect of C1orf64 silencing on the DHT-mediated induction of PIP expression in MFM-223 cells as outlined in **(C).** **(E)** immunoblotting to assess C1orf64 protein levels in T-47D and MFM-233 cell lines following C1orf64 overexpression. Fold change (RR) in each band density was measured relative to control in three replicate experiments. CT: control vector, OE: overexpression. **(F)** qRT-PCR to assess the effect of C1orf64 overexpression on PIP expression in T-47D and MFM-223 cells. CT-VEC: Control vector, C1orf64+: C1orf64 overexpression. *, p≤ 0.01 is for CT-VEC vs. C1orf64+.

To investigate the effect of C1orf64 on PIP transcription, silencing of C1orf64 was carried out using two siRNA duplexes (siRNA-D1 and siRNA-D2) and transfections with a non-targeting siRNA were used as a control (CTL). The efficiency of C1orf64-knockdown was then assessed by calculating the fold changes in C1orf64 expression using qRT-PCR seventy-two hours after siRNA transfections. Notably, C1orf64 expression was down-regulated by over 95% using siRNA-D1 and siRNA-D2 in both T-47D and MFM-223 cell lines (Figure 5A). Next, the effect of C1orf64 expression on the baseline levels of PIP transcription was examined following C1orf64-siRNA transfections in T-47D and MFM-223 grown in the full media. Importantly, there was a 6 to 7-fold increase in PIP expression following C1orf64 silencing in T-47D cells with both siRNA duplexes (p< 0.01, Figure 5B). In addition, C1orf64 silencing increased PIP expression by approximately 3 to 5-fold in MFM-223 cell line (p< 0.01, Figure 5B). Moreover, the effect of C1orf64 silencing with siRNA-D1 was examined on PIP protein level in T-47D cell line, which has a measurable level of PIP protein using western blotting on cell lysates [14]. Consistent with the mRNA data, C1orf64 silencing resulted in a marked increase in PIP protein level by over 10-fold (Supplementary Figure 2A). These findings indicate that C1orf64 has a profound repressive effect on PIP expression.

Furthermore, the effect of C1orf64 on DHT-mediated induction of PIP expression was investigated in T-47D and MFM-223 cell lines. C1orf64-silenced cell lines were grown in phenol red-free media supplemented with 10% Charcoal/Dextran treated serum for 48h and then DHT treatment was carried out at 10 nM concentration for 24h. Fold changes in PIP expression were measured using qRT-PCR relative to the control experiments. In T-47D cells, there was an 11.8-fold increase in PIP expression following DHT treatment compared to control (p <0.01, Figure 5C). In addition, C1orf64 silencing further enhanced the DHT-mediated induction of PIP expression compared to control by 16.4-fold and 43-fold for siRNA-D1 and siRNA-D2, respectively (p< 0.03 for DHT + control (CTL)-siRNA vs. DHT + siRNA-D1 or siRNA-D2, Figure 5C). Moreover, in MFM-223 cells, DHT treatment induced PIP expression by 2-fold (p< 0.01, Figure 5D), and C1orf64 silencing further increased PIP expression by 4.5-fold compared to control (p< 0.03 for DHT + CTL-siRNA vs. DHT + siRNA-D1 or siRNA-D2, Figure 5D). Therefore, C1orf64 expression represses the DHT-mediated induction of PIP.

Next, the level of PIP expression was examined following C1orf64 silencing in combination with enzalutamide treatment. Enzalutamide is an AR inhibitor that is used in both research and clinical practice [18]. Forty-eight hours after the transfections of T-47D and MFM-223 cells with siRNA-D1, enzalutamide treatment was carried out at 10 μM concentration for 24h followed by RNA extraction and measurement of PIP and C1orf64 expression using qRT-PCR. Cells transfected with CTL-siRNA and treated with enzalutamide or solvent only were used as control experiments. Next, fold changes in gene expression were assessed relative to CTL-siRNA/solvent-treated group. Consistent with the previous experiments, there was over 95% reduction in C1orf64 expression following siRNA silencing in both cell lines (Supplementary Figure 2B). In addition, enzalutamide treatment significantly reduced PIP expression in T-47D and MFM-223 cells by 50% and 13%, respectively (p<0.01, Supplementary Figure 2B). Importantly, C1orf64 silencing reversed the effect of enzalutamide on PIP transcription in siRNA-D1/enzalutamide group and led to an increase in PIP expression by 9.95 and 1.34-fold in T-47D and MFM-223 cell lines, respectively (p< 0.01, Supplementary Figure 2B). These findings suggest that C1orf64 expression is required for the enzalutamide-mediated down-regulation of PIP expression.

Finally, the effect of C1orf64 overexpression on PIP transcription was examined using the transfection of a C1orf64 expression construct in T-47D and MFM-223 cell lines. Transfection with an empty plasmid was applied for the control experiments. Cell lines were grown in the presence of DHT and the effect of C1orf64 overexpression was assessed 48h following transfections. The overexpression efficiencies were evaluated using western blot analysis to measure C1orf64 protein levels in C1orf64-overexpressed and control cell lines. Notably, there was a marked increase in C1orf64 protein levels by approximately 100-fold in the overexpressed cells compared to the control experiments (Figure 5E). Next, the effect of C1orf64 overexpression was assessed on PIP expression using qRT-PCR. Importantly, PIP expression was moderately decreased by 1.43 to 1.67-fold following C1orf64 overexpression in MFM-223 and T-47D cells (p ≤ 0.01, Figure 5F). Therefore, the presented data for overexpression and silencing experiments suggest that C1orf64 has a repressive effect on PIP expression and the DHT-mediated induction of PIP in breast cancer cells.

### C1orf64 silencing intensifies the AR-mediated repression of PGR

**Figure 6:**
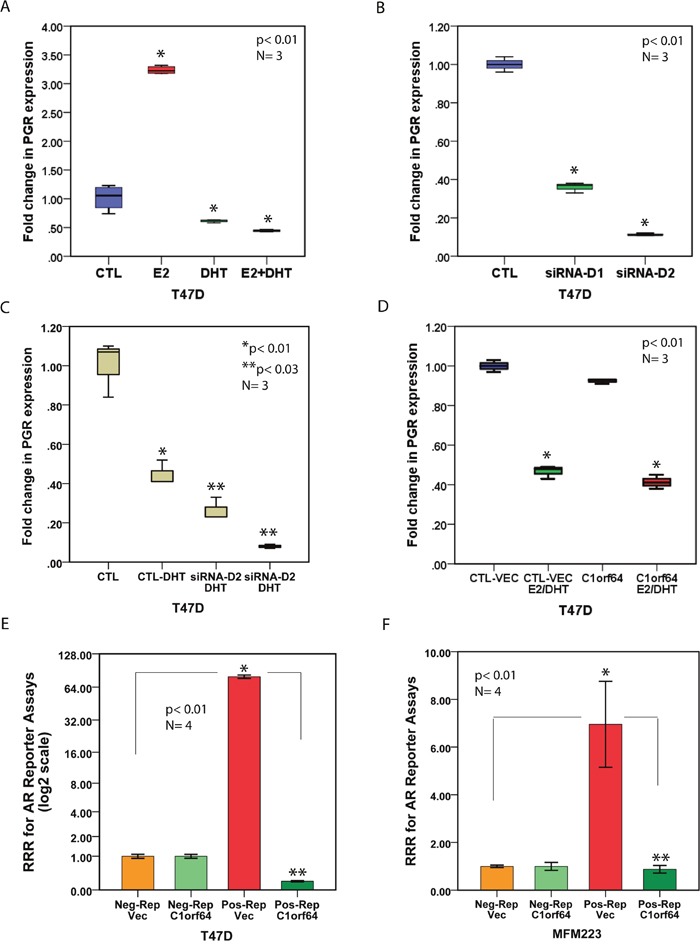
The effect of C1orf64 on the AR-mediated repression of PGR and AR reporter activity **(A)** qRT-PCR to assess the effects of E2 and DHT on PGR expression in T-47D cell line. CTL: solvents only, E2: E2 at 10 nM, DHT: DHT at 10 nM, E2 + DHT: each at 10 nM. *, p< 0.01 is for each treatment group vs. CTL. **(B)** qRT-PCR to measure the effect of C1orf64 silencing using siRNA-D1 (D1) and siRNA-D2 (D2) on PGR expression in T-47D cells. CTL: control-siRNA. *, p< 0.01 is for CTL vs. D1 or D2 **(C)** qRT-PCR to examine the combined effects of DHT (10 nM) treatment and C1orf64 silencing on PGR expression. CTL: CTL-siRNA, *, p< 0.01 is for CTL vs. CTL-DHT, and **, p< 0.03 is for CTL-DHT vs. siRNA-D1 + DHT or siRNA-D2 + DHT. **(D)** qRT-PCR to assess the effect of C1orf64 overexpression on PGR expression. CTL-VEC: Control vector, E2/DHT: E2 (10 nM) + DHT (10 nM), C1orf64: C1orf64 overexpression. *, p< 0.01 is for CTL-VEC vs. CTL-VEC + E2/DHT or C1orf64 vs. C1orf64 + E2/DHT. **(E-F)** AR reporter assays in T-47D **(E)** and MFM-223 **(F)** cell lines. Relative response ratio (RRR) is shown for each reporter assay. Neg-Rep/Vec: negative control for reporter + empty plasmid, Neg-Rep/C1orf64: negative control for reporter + C1orf64 construct, Pos-Rep/Vec: AR reporter + empty plasmid, and Pos-Rep/C1orf64: AR reporter + C1orf64 construct. *p<0.01 is for Pos-Rep/Vec vs. Neg-Rep/Vec, **p< 0.01 is for Pos-Rep/C1orf64 vs. Pos-Rep/Vec or Neg-Rep/C1orf64. Error Bars: ± 2SEM.

To further evaluate the function of C1orf64 on AR transcriptional activity, the effect of this gene was examined in the process of AR-mediated gene repression. It is known that AR inhibits endogenous ERα transactivation in ER-positive breast cancer [28]. In view of this, the effect of AR activation was assessed on the expression of an established ER target gene, progesterone receptor (PGR), [29, 30]. T-47D cell line was utilized for these experiments due to its ER-positive status. Treatments were carried out in the following groups: E2 at 10 nM, DHT at 10 nM, and E2 (10 nM) + DHT (10 nM). Control experiments were performed by adding the respective solvent of each compound and PGR expression levels were measured 24h after treatments using qRT-PCR. As expected, E2 treatment led to a significant increase in PGR expression by 3.2-fold (p< 0.01, Figure 6A). Notably, DHT significantly reduced PGR expression by 2-fold (p< 0.01. Figure 6A). Moreover, when combined with E2, DHT fully reversed the stimulatory effect of E2 on PGR expression and further down-regulated its expression by 2-fold to a level similar to that observed with the DHT treatment alone (p< 0.01, Figure 6A). Therefore, AR activation represses PGR expression and inhibits the stimulatory effect of E2 on this gene.

Furthermore, the effect of C1orf64 silencing on the baseline expression of PGR was evaluated. To achieve this, C1orf64 was silenced in T-47D cells with siRNA-D1 and siRNA-D2 and PGR expression was assessed using qRT-PCR. There was a significant reduction in PGR expression following C1orf64 silencing by 2.8-fold and 9.1-fold for siRNA-D1 and siRNA-D2, respectively, indicating that C1orf64 is necessary for PGR expression (p< 0.01, Figure 6B). Next, the combined effects of DHT treatment and C1orf64 silencing on PGR expression were examined. In this process, T-47D cells were evaluated in the following experimental groups: CTL-siRNA, CTL-siRNA + DHT, siRNA-D1 + DHT, and siRNA-D2 + DHT. As previously noted, DHT treatment reduced PGR expression by approximately 2-fold (p< 0.01, Figure 6C). Importantly, C1orf64 silencing significantly intensified the DHT-mediated repression of PGR by 3.85-fold and 12.5-fold for siRNA-D1 and siRNA-D2, respectively (p< 0.03 for DHT + CTL-siRNA vs. DHT + siRNA-D1 or siRNA-D2, Figure 6C).

To assess whether C1orf64 overexpression can reverse the repressive effect of DHT on E2-mediated induction of PGR expression, T-47D cells were transfected with a C1orf64 expression construct or an empty plasmid. In addition, transfected cells were either treated with the combination of E2 (10 nM) + DHT (10 nM) or with the solvents only. Consistent with the previous experiments, E2/DHT treatment reduced PGR expression by 2.2-fold in the control vector-transfected group and a similar level of decrease in PGR expression was also observed following E2/DHT treatment in C1orf64-overexpressed cells (p< 0.01, Figure 6D). Moreover, C1orf64 overexpression did not significantly change the baseline level of PGR expression (Figure 6D). These findings indicate that although C1orf64 silencing intensifies the DHT-mediated repression of PGR, C1orf64 overexpression cannot overcome the reduction in PGR transcription resulted from AR activation.

### C1orf64 overexpression inhibits AR reporter activity

Moreover, the effect of C1orf64 expression was investigated on AR reporter activity using luciferase assays in T-47D and MFM-223 cell lines. In this process, co-transfections were carried out in the following four experimental groups as explained in methods: 1) Negative control for reporter + empty plasmid (Neg-Rep/Vec), 2) Negative control for reporter + C1orf64 expression construct (Neg-Rep/C1orf64), 3) AR reporter + empty plasmid (Pos-Rep/Vec), and 4) AR reporter + C1orf64 expression construct (Pos-Rep/C1orf64). Twenty-four hours after co-transfections, all groups underwent a change of media that included DHT at 10 nM concentration followed by the measurement of reporter activities at the 48h time-point. Finally, RRR was calculated for each experimental group as described in methods. As expected, there was a marked induction of AR reporter activity in Pos-Rep/Vec groups by 80 (± 2.8) and 7 (±1.8)-fold in T-47D and MFM-223 cell lines, respectively (p< 0.01, Figure 6E and 6F). Importantly, C1orf64 overexpression completely inhibited the AR reporter activities in both cell lines as demonstrated by a full reversal of RRR inductions observed in Pos-Rep/Vec groups compared to that of Pos-Rep/C1orf64 experiments (p< 0.01, Figure 6E and 6F). In addition, there was a 5-fold decrease in RRR of Pos-Rep/C1orf64 group compared to that of Neg-Rep/C1orf64 in T-47D cells, which collectively amounts to a total of 400-fold reduction in AR reporter activity following C1orf64 overexpression in this cell line (p< 0.01, Figure 6E). Taken together, these findings indicate that C1orf64 has a profound repressive effect on the AR reporter activity.

### C1orf64 silencing increases AR binding to the PIP promoter

**Figure 7:**
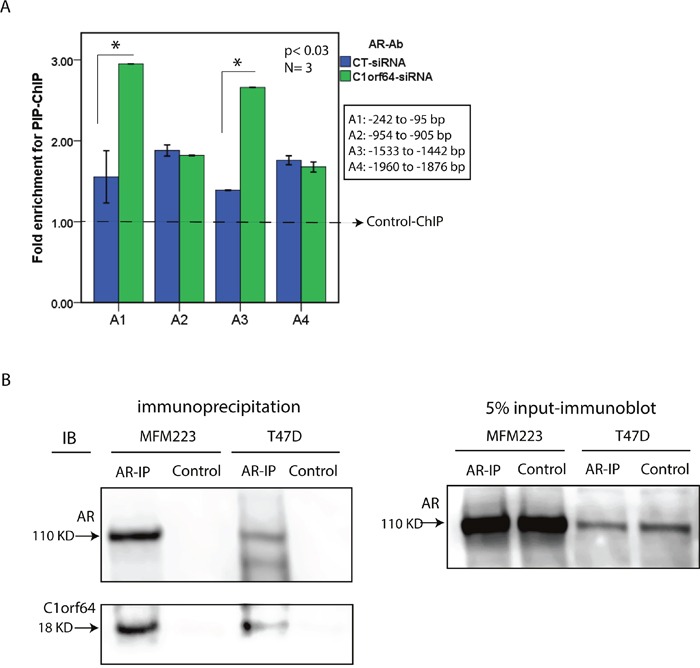
The effect of C1orf64 on AR promoter binding and the interaction between C1orf64 and AR **(A)** Fold enrichments of ChIP for PIP promoter performed with an AR antibody (AR-Ab) following the transfections of control (CT)-siRNA or C1orf64-siRNA (Duplex 1) in T-47D cells. AR binding to each promoter region (A1-A4) was assessed. Amplification of 1% of input chromatin was used as the input control and a non-specific antibody served as negative control (dashed-line). *, p< 0.03 is for C1orf64-siRNA vs. CT-siRNA in A1 and A2. Error Bars: ± 2SEM. **(B)** Co-immunprecipitation assays (Co-IP) to investigate the interaction between endogenous C1orf64 and AR in T-47D and MFM-223 cell lines. AR-IP was performed using an AR antibody and control experiments were conducted with a non-specific rabbit IgG. Immunoblot analysis were carried out on IP supernatants using C1orf64 and AR antibodies. Input was assessed with AR immunoblotting on 5% of lysates collected before each IP. All co-IP experiments were performed in three replicates.

The observation that C1orf64 represses the AR-mediated induction of PIP, raises the question of a possible regulatory function for C1orf64 in AR binding to the PIP promoter. This possibility was investigated using the transfection of T-47D cells with siRNA-D1 or CTL-siRNA followed by ChIP assays for PIP promoter. Forty-eight hours after siRNA transfections, T-47D cells were treated with 10 nM of DHT for 24h and ChIP assays were performed using AR antibody and four primer sets in the 2 kb region of PIP promoter. A total of three replicates were carried out for each experiment and fold enrichments for ChIP-signal were calculated using qRT-PCR as explained in methods. Importantly, C1orf64 silencing significantly increased the enrichment for AR binding to PIP promoter in the regions of A1 (-242 to -95 bp) and A3 (-1533 to -1442) by approximately 2-fold (p< 0.03, Figure 7A). However, the A2 and A4 regions of PIP promoter did not demonstrate a significant change in AR binding after C1orf64 silencing (Figure 7A). These data suggest that C1orf64 expression has a repressive function on AR binding to the PIP promoter.

### C1orf64 interacts with AR in breast cancer cells

The repressive function of C1orf64 on AR transcriptional activity suggests that C1orf64 acts a negative regulator of AR function in breast cancer cells. Therefore, to examine whether C1orf64 is a coregulator of AR, the protein-protein interaction between endogenous C1orf64 and AR was studied in T-47D and MFM-223 cell lines using co-IP assays. Following immunoprecipitation (IP) assays with an AR antibody, supernatants were collected and applied for immunoblot analysis to examine the pull-down of C1orf64 and AR proteins. Control assays were conducted with a non-specific rabbit IgG and all experiments were performed in three replicates. In addition, 5% of lysates were collected before each IP to assess input using AR immunoblotting (Figure 7B). Importantly, immunoblot analysis for C1orf64 antibody detected a distinct band for C1orf64 protein in the AR-IP samples that was present in both T-47D and MFM-223 cell lines (Figure 7B). It is notable that a C1orf64 band was not present in the control-IP experiments (Figure 7B). Furthermore, immunoblotting with an AR antibody confirmed a successful pull-down of AR following IP assays in both cell lines and as expected showed a higher level of AR in MFM-223 cells compared to T-47D line (Figure 7B). These findings indicate that C1orf64 interacts with AR in breast cancer cells.

### C1orf64 interacts with 14-3-3 protein

**Figure 8:**
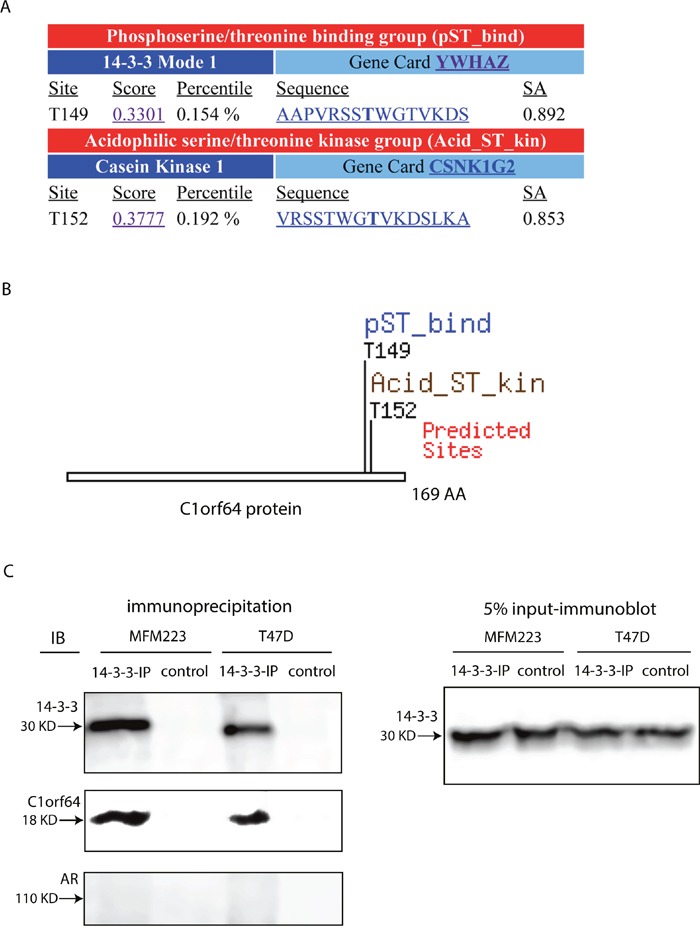
An interaction between C1orf64 and 14-3-3 **(A and B)** C1orf64 protein sequence was analyzed using Scansite software to identify binding sites for regulatory motifs. Motif scan was carried out with a high stringency to detect the best 0.2% of all sites. **(A)** Shows each motif, sequence score with percentile, sequence of motif, and surface accessibility of the predicated site. **(B)** Depicts the motif sites within C1orf64 sequence. **(C)** Co-immunprecipitation assays to examine the interaction between endogenous 14-3-3 and C1orf64 as well as with AR in T-47D and MFM-223 cell lines. IP assays were performed using a 14-3-3 antibody and control experiments were conducted with a non-specific rabbit IgG. Immunoblot analysis were carried out on IP supernatants using 14-3-3, C1orf64, and AR antibodies. Input was assessed with 14-3-3 immunoblotting on 5% of lysates. All co-IP experiments were performed in three replicates.

To further explore the molecular mechanisms of C1orf64 function, the sequence of this protein was examined using Scansite software as explained in methods to identify motifs that are likely to be phosphorylated by specific protein kinases or bind to domains such as SH2 domains, 14-3-3 domains or PDZ domains. This search, which was carried out with a high stringency to detect the best 0.2% of all sites, identified two motifs within C1orf64 sequence. The first motif was 14-3-3 Mode 1, which is a phosphoserine/threonine binding group (pST_bind), and was predicted to interact with C1orf64 at T149 site with a motif score of 0.3301 in the top 0.154% of all sites (Figure 8A and 8B). The predicated gene for this motif is YWHAZ (14-3-3 Zeta) that can interact with AAPV**RSSTWG**TVKDS sequence within C1orf64 protein and the underlined region depicts a consensus sequence for 14-3-3 binding. The other predicated motif was Casein Kinase 1, an acidophilic serine/threonine kinase, which can interact with C1orf64 at T152 site with a score of 0.3777 in the top 0.192% of all sites (Figure 8A and 8B). Together, these findings suggest that C1orf64 is a phosphothreonine protein, which is phosphorylated by Casein Kinase 1 and interacts with 14-3-3 in an adjacent motif.

It is notable that 14-3-3 is known to interact with various steroid receptors and has a regulatory function on these proteins [31–33]. Therefore, the predicated interaction between C1orf64 and 14-3-3 was further investigated using co-IP of endogenous proteins in T-47D and MFM-223 cell lines. In this process, following IP assays for 14-3-3, supernatants were collected and applied for immunoblotting to examine the pull-down of 14-3-3, C1orf64, and AR proteins. Control assays were conducted with a non-specific rabbit IgG. In addition, 5% of lysates were collected before each IP to assess input using 14-3-3 immunoblotting (Figure 8C). Notably, immunoblotting with the 14-3-3 antibody confirmed a successful IP of 14-3-3 protein (Figure 8C). Furthermore, immunoblot analysis using C1orf64 antibody detected a distinct band for C1orf64 in the 14-3-3 IP assays that was present in both cell lines (Figure 8C). In addition, C1orf64 band was absent in the control-IP experiments (Figure 8C). However, there was no detectable interaction between 14-3-3 and AR in these cell lines (Figure 8C). These findings indicate that C1orf64 interacts with 14-3-3 in breast cancer cells.

## DISCUSSION

AR is widely expressed in both ER-positive and ER-negative breast tumors [1–3]; however, the molecular functions of AR in breast cancer are not well understood. Therefore, this study investigated the network of genes that are co-expressed with AR in order to discover novel AR targets with biological significance in breast cancer. In this process, bioinformatics analysis of two large datasets from a broad range of breast cancer cell lines resulted in the identification of a combined AR-gene signature constituted of 98 genes that highly correlated with AR expression (Table 1). In addition, approximately 75% of the signature genes, which had an overlap between the two datasets, significantly correlated with AR expression in both cohorts. This indicates a high level of reproducibility in signature genes despite the differences between the two platforms. For instance, F7 that was previously identified as an AR target gene [20], highly correlated with AR expression in both datasets. Furthermore, functional annotation of the AR-gene signature revealed that it is highly enriched for genes related to DNA binding and transcription factor binding, suggesting that this signature is closely associated with the established molecular functions of AR transcriptional network. Therefore, the study of AR-signature genes would provide a valuable approach to better understand the molecular functions of AR in breast cancer.

Notably, C1orf64 showed the closest correlation with AR expression across the datasets with a CC of 0.737 and it also highly correlated with AR expression in both primary and metastatic breast tumors with CC values of 0.667 and 0.573, respectively (Table 1 and Figure 4). There is very limited information about the molecular and biochemical functions of C1orf64 with only a few published studies available on this gene using an alias identifier, ERRF [34–36]. These studies showed that C1orf64 is expressed in breast tumors with a higher expression level in ER-positive cancers [34], a finding that was also confirmed in the current study (Table 4). In addition, there is some evidence to suggest that C1orf64 is involved in the transcriptional activities of ER and there is an interaction between ER and C1orf64 using the transient transfection of cells with tagged C1orf64 and ER constructs [35]. Furthermore, a recent study has demonstrated that ER activation results in the suppression of C1orf64 expression in ER-positive cells; however, authors did not observe any evidence for a direct ER binding to the promoter of C1orf64 [36]. These findings suggest that estrogen-mediated down-regulation of C1orf64 may not be a direct effect. Therefore, the published data indicate that C1orf64 plays a role in the ER transcriptional activities; however, they do not provide information about the broader activities of this protein such as in ER-negative tumors and the biochemical mechanisms of C1orf64 function.

The data presented here show a strong association between AR and C1orf64 expression in breast cancer that is not limited to ER-positive tumors. In fact, C1orf64 is expressed at a slightly higher level in ER-/AR+ MFM-223 cells compared to ER+/AR+ T-47D line (Figure 3A). However, it is also notable that 70% of breast cancers are ER-positive and AR is expressed in 90% of this subgroup compared to 50% of ER-negative tumors [1–3]. Therefore, this differential pattern of AR expression can explain a relatively higher expression of C1orf64 observed in ER-positive compared to ER-negative tumors and at the same time a strong correlation between AR and C1orf64 expression across all breast cancer subtypes. Furthermore, C1orf64 has a relatively higher expression in lower grade tumors, lobular histology, and non-TN biomarker groups. These findings agree with each other since it is known that lobular tumors tend to be of a lower grade and ER-positive status, although their outcome are generally similar to that of ductal tumors [37].

**Figure 9:**
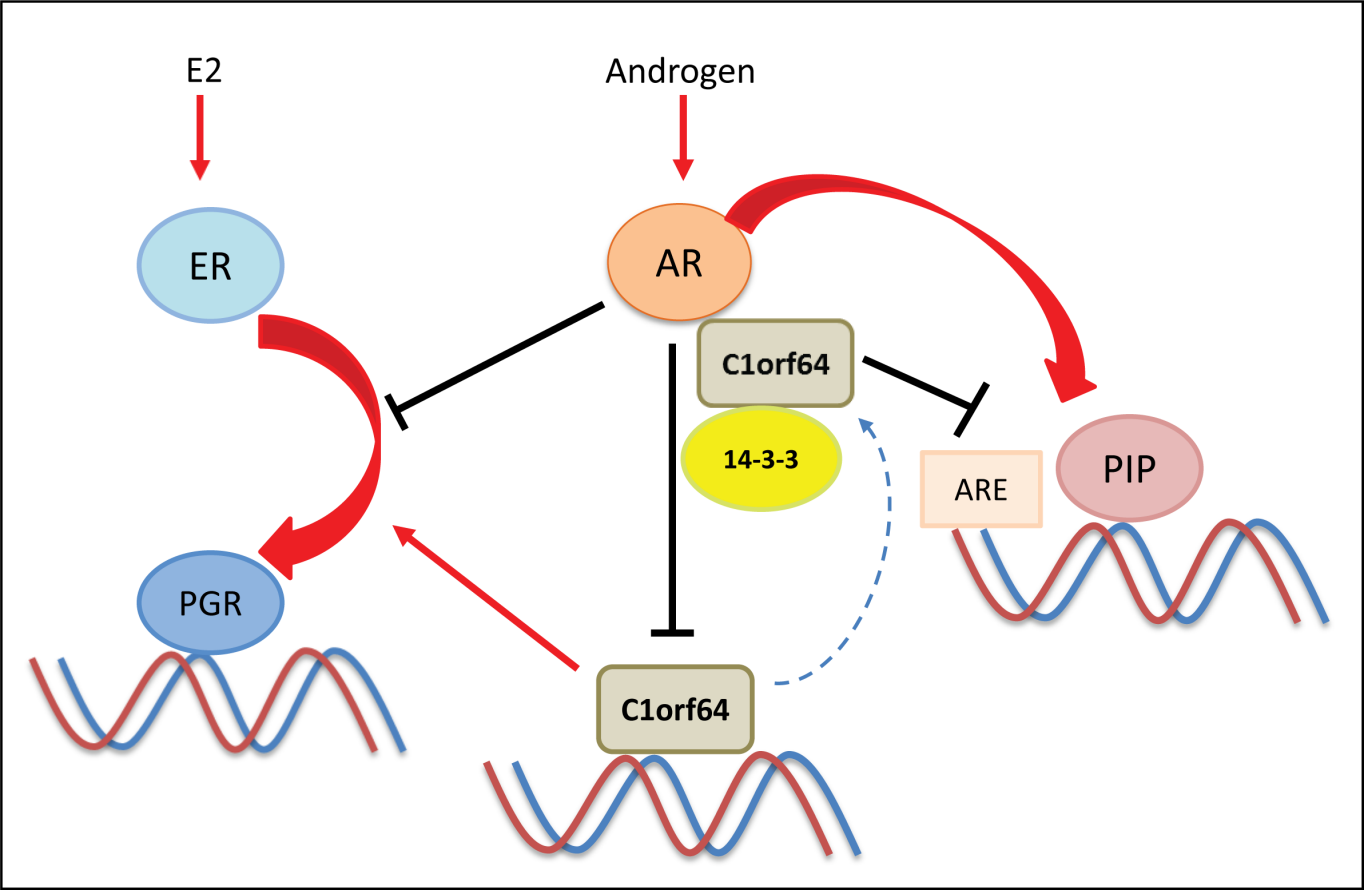
A schematic model for the interplay between AR and C1orf64 This model shows a negative interplay between AR and C1orf64. In this process, AR activation represses C1orf64 transcription and C1orf64, in turn, acts as a corepressor of AR and negatively regulates the AR-mediated induction of PIP expression and AR reporter activity. Chaperone protein 14-3-3 interacts with C1orf64 and has a regulatory function in this model. The other aspect of this interplay involves a cross-talk between the AR and ER signaling in which, AR activation has a repressive effect on the ER-mediated induction of PGR. In addition, C1orf64 is necessary for PGR expression and its repression by AR has an inhibitory effect on the positive regulation of ER activity by C1orf64. Red arrows denote a stimulatory effect and black lines indicate a repressive function.

Importantly, there is a functional interplay between AR and C1orf64 in breast cancer cells (Figure 9). In this process, AR activation directly represses C1orf64 transcription and C1orf64, in turn, interacts with AR as a corepressor and negatively regulates the AR-mediated induction of PIP expression (Figure 9). This negative regulation is further supported by the fact that C1orf64 has a profound repressive effect on the AR reporter activity in breast cancer cells (Figure 6E and 6F). In addition, the corepressor effect of C1orf64 results in a reduction of AR binding to PIP promoter. Furthermore, the other aspect of this interplay involves a cross-talk between the AR and ER signaling in ER-positive cells. In this respect, AR activation has a repressive effect on the ER-mediated induction of PGR. In view of the fact that C1orf64 is necessary for PGR expression, the repression of C1orf64 by AR has an inhibitory effect on the positive regulatory effect of C1orf64 on ER activity (Figure 9). It is notable that C1orf64 is required for the enzalutamide-mediated down-regulation of PIP expression. This finding is consistent with the recent observations that AR corepressor N-CoR1 levels decline with the progression of prostate cancer and this process is involved in the development of resistance to bicalutamide treatment [38]. Therefore, in a similar way, C1orf64 may have a function in mediating the activities of AR inhibitors in breast cancer.

This study suggests that C1orf64 is a phosphothreonine protein that interacts with 14-3-3 and is phosphorylated by Casein Kinase 1 at a T152 site adjacent to the 14-3-3 interacting motif (Figure 8). Notably, 14-3-3 is a chaperone and scaffolding protein that binds serine/threonine-phosphorylated residues and regulates key proteins involved in various cellular processes such as intracellular signaling and gene transcription [33, 39–42]. In view of the fact that 14-3-3 only interacts with phosphorylated proteins, the presence of a Casein Kinase I binding site in an adjacent region to that of 14-3-3 motif, would facilitate the interaction between C1orf64 and 14-3-3 proteins. In addition, these findings suggest that the C-terminal region of C1orf64 is involved in key protein interactions. Interestingly, it has been shown that 14-3-3 interacts with different steroid receptors and mediates both positive and negative regulatory effects on these receptors [31–33]. In this respect, it has been suggested that 14-3-3 interaction with AR promotes the transcriptional activation of PSA promoter in prostate cancer cells [40]. Conversely, 14-3-3 interaction with ERα in breast cancer cells negatively affects ER/DNA interaction, ER transactivation activity, and ER-dependent cell growth [41]. Moreover, 14-3-3 interaction with glucocorticoid receptor (GR) enhances GR transactivation by binding and removing its corepressor RIP140 [42].

However, this study did not show a direct interaction between endogenous 14-3-3 and AR in breast cancer cells. By and large, most studies for 14-3-3 interaction with steroid receptors were conducted either using non-cell based assays such as yeast two-hybrid screens or by the application of tagged overexpression constructs, with the exception of ER/14-3-3 interaction that was examined on endogenous proteins in MCF-7 cell line [31–33]. Therefore, there is limited information about the interaction of endogenous 14-3-3 with steroid receptors in various tissues. In addition, the interactions between endogenous proteins can be transient and tissue specific. In view of these points, a plausible mechanism for C1orf64 coregulatory effects on AR and ER can involve a competition with 14-3-3 binding to these receptors or acting as a scaffolding protein in a complex that includes 14-3-3 and a steroid receptor tethering them together (Figure 9).

It is notable that PIP is a key molecular target of AR that is widely expressed in luminal A, luminal B, and molecular apocrine subtypes of breast cancer and is necessary for cell cycle progression in breast cancer cells [14]. Therefore, the repressive effect of C1orf64 on PIP expression would have an impact on the AR-PIP signaling pathway. However, the overall cellular activities of C1orf64 are also influenced by the net regulatory functions of this protein on other AR and ER-regulated genes such as PGR. Importantly, both silencing and overexpression experiments demonstrated that C1orf64 has a repressive function on PIP expression as well as the DHT-mediated induction of PIP (Figure 5). This is a reflection of the fact that AR activation is necessary and sufficient for PIP expression in breast cancer cells [8]. In comparison, although C1orf64 silencing intensifies the DHT-mediated repression of PGR, the overexpression of this gene cannot reverse the inhibitory effect of DHT on E2-mediated PGR activation (Figure 6). These data indicate that AR regulation of C1orf64 is not the only mediator of the AR-ER cross-talk. In fact, the mechanisms for the inhibition of ER activity by AR in breast cancer are not fully understood and one suggested mechanism is direct AR binding to a subset of estrogen response elements that can prevent E2-mediated activation of ER target genes [28]. Therefore, AR repression of C1orf64 provides another mechanism for the AR inhibitory effects on ER signaling in breast cancer cells (Figure 9).

The presented data suggest that C1orf64 is a novel AR coregulaor, which represses the AR transcriptional effects on PIP and PGR expression, and inhibits the AR reporter activity. Intriguingly, since C1orf64 can regulate both AR and ER activities in opposite directions, it may be a bifunctional coregulator that acts as a corepressor or a coactivator depending on the interacting transcription factors and the regulated genes. Notably, a number of bifunctional (two-faced) coregulators have been identified so far that include classical coregulators SRC1 and N-CoR/SMRT as well as other coregulators such as RIP140, NSD1 and PELP1, which can function as either a coactivator or a corepressor for nuclear receptors [43–45]. Therefore, further studies are required to better understand the coregulatory functions of C1orf64 on other nuclear receptors and in different tissue types. The positive correlation of AR and C1orf64 expression in breast cancer can be explained by the fact that in order for AR and its coregulators to function together, both must be expressed in the same cells. The negative interplay between AR and C1orf64 suggests a biological basis for this coexpression to maintain a balanced AR activity within the cell. In fact, a positive correlation between the expression of steroid receptors and their corepressors have been previously reported. For example, ER-α expression positively correlates with the expression of N-CoR and SMRT in endometrial cancer and with N-CoR expression in breast cancer [46, 47]. Finally, in view of the fact that C1orf64 is regulated by both AR and ER, it would be appropriate to propose naming this protein, “Steroid Receptor-Regulated Protein” or SRRP.

Moreover, in this study SPDEF (Sam-pointed domain containing Ets transcription factor) was also identified as an AR target gene that is induced by DHT in MFM-223 cells. SPDEF belongs to the Ets family of transcription factors that is expressed in luminal, apocrine, and ErbB2-positive breast tumors and is associated with poor prognosis [48]. In addition, SPDEF can drive luminal differentiation of basal mammary epithelial cells, regulate the survival of luminal tumor cells, and contribute to endocrine resistance [49]. It is notable that, as opposed to the DHT induction of SPDEF in MFM-223 cells, there was a slight reduction in SPDEF expression following DHT treatment in T-47D cells. These findings may be related to the differences between ER-positive and ER-negative cells with respect to the effect of other transcription factors on AR regulation of SPDEF expression. In fact, it has been shown that in ER-positive cells, GATA3 and FOXA1 both modulate the ER-mediated regulation of SPDEF transcription [49]. Interestingly, C1orf64 shows a strong correlation with SPDEF expression in breast cancer at a level similar to that observed with AR (Table 3 and Figure 4). In addition, C1orf64 is closely co-expressed with over 60% of the AR-signature genes that have a detectable AR binding in ChIP-seq data (Table 3). These findings suggest that C1orf64 may broadly take part in the AR transcriptional activities in breast cancer and participate in a transcriptional network with the other AR-signature genes such as SPDEF.

In summary, this study investigated the network of genes that are co-expressed with AR in breast cancer and identified C1orf64 as a novel AR target gene and coregulator in this disease. The presented data demonstrate that AR activation directly represses C1orf64 transcription in breast cancer cells. In turn, C1orf64 interacts with AR as a corepressor and negatively regulates the AR-mediated effects on PIP and PGR expression, and AR reporter activity. Importantly, C1orf64 also interacts with the chaperone protein 14-3-3, which can provide an underlying mechanism in mediating the molecular functions of C1orf64 by modulating the chaperone functions of this protein. All together, these data suggest an interplay between C1orf64 and AR with significance in the biology of breast cancer. Intriguingly, in view of the fact that C1orf64 regulates the transcriptional functions of both AR and ER, exploring the modulation of C1orf64 may provide a novel therapeutic strategy for targeting the steroid receptor signaling in hormone-sensitive malignancies.

## MATERIALS AND METHODS

### Bioinformatics

#### Gene expression analysis

Two expression microarray datasets were extracted from the studies published by Neve *et al*. and Kao *et al*. [21, 22]. Both datasets constituted of widely used ER-positive and ER-negative breast cancer cell lines. The first study (study 1) was conducted on a cohort of 52 breast cancer cell lines by Neve *et al*. and was also applied in a previous publication by my group [20, 21]. This study included a total of 22,216 gene expression data points for each cell line using Affymetrix highdensity oligonucleotide array human HG-U133A chip [21]. The second dataset (study 2) was extracted from a study performed on a cohort of 50 breast cancer cell lines using Human Exonic Evidence Based oligonucleotide (HEEBO) arrays obtained from the Stanford Functional Genomics Facility that contained 36,192 oligonucleotides representing 18,141 mapped human genes [22]. It is notable that these studies had a total of 37 overlapping cell lines and a total of 28 cell lines were varied between the two cohorts [21, 22].

The extracted gene expression matrix from each study was analysed to identify genes that were highly co-expressed with AR across each dataset. In this respect, Pearson correlation coefficients (CC) between AR expression and that of every gene in each dataset were calculated. Next, a list of highly co-expressed genes with AR was generated in each dataset using a cutoff of |CC| ≥ 0.6, p< 0.001. For study 1, a list of 35 highly co-expressed genes with AR was obtained from the analysis performed in my previous publication [20]. Following the analysis of study 2, a second list of highly co-expressed genes with AR was identified and used to generate a combined “AR-gene signature” by compiling these two gene sets across both cohorts (|CC|≥ 0.6, p< 0.001). In addition, genes that were present in both microarray platforms and were also significantly co-expressed in both datasets with a p< 0.05 were identified. Data analysis to obtain CC values was performed using IBM SPSS Statistics 23 (Armonk, NY, USA).

Furthermore, molecular functions of the combined AR-gene signature were assessed using functional annotation as described before [20]. To achive this, gene symbols of the combined AR-signature were first updated to the official symbols provide by Hugo Gene Nomenclature Committee [50]. Next, bioinformatics analysis of this gene signature was performed to obtain Gene Ontology (GO) and protein annotations in two categories of GOTERM and INTERPRO using The Database for Annotation, Visualization and Integrated Discovery (DAVID) Bioinformatics Resources v6.8 (National Institute of Allergy and Infectious Diseases, Bethesda, MD, USA), [51, 52]. It is notable that GOTERM predicts GO annotation and INTERPRO provides functional analysis of proteins by classifying them into families [51, 52].

#### Co-expression analysis in breast tumors

*ONCOMINE 4.5* database was used to identify genes that highly correlate with C1orf64 expression in breast tumors (www.oncomine.org), [23]. In this process, co-expression analysis for C1orf64 was carried out in two large breast tumor datasets. The first dataset included The Cancer Genome Atlas (TCGA)-Invasive Breast Carcinoma Gene Expression Data performed by the Office of Cancer Genomics, National Cancer Institute, National Institutes of Health (https://gdc.cancer.gov/), [24]. This cohort contains a total of 593 samples with 532 invasive breast carcinoma and 61 paired normal breast tissue. In addition, 3 paired metastatic samples were analyzed. In this study, a total of 20,423 genes were assessed for each sample. The second dataset was obtained from a study conducted by Bos *et al*. at Memorial Sloan-Kettering Cancer Center on genes that mediate breast cancer metastasis to the brain [25]. This cohort consists of 204 metastatic breast carcinoma samples and expression profiling was performed using Human Genome U133 Plus 2.0 Affymetrix Arrays measuring 19,574 genes [25]. Co-expression analysis to identify genes that are highly correlated with C1orf64 expression (p≤ 0.0001) was carried out using *ONCOMINE* and CC values and heat maps of C1orf64 co-expressed genes were obtained for each dataset.

#### Analysis of C1orf64 association with clinical and pathological features

First, a differential analysis was carried out using *ONCOMINE 4.5* for breast cancer datasets with available clinical and pathological features (www.oncomine.org), [23]. In this process, C1orf64 log2 median expression values were obtained for each of the pathological and clinical subtypes that were available across twenty-two breast cancer datasets. These included data for histology type, tumor grade, ER status, ErbB2 status, triple negative (TN) status, and outcome. Next, to identify differential expression patterns, C1orf64 log2 median values for each subtype of the clinical and pathological features were compared using Wilcoxon Signed Rank Test. P values were calculated for the following comparisons: ductal vs. lobular; grade 1 vs. grade 3; grade 2 vs. grade 3; ER-negative (Neg) vs. ER-positive (Pos); ErbB2-neg vs. ErbB2-pos; TN vs. other biomarker status; good outcome (good: no recurrence or death in 5 years) vs. poor (recurrence or death in 5 years). Finally, fold difference in C1orf64 expression was calculated for each significant p value.

#### Promoter analysis

Identification of predicated AR binding sites in the 2 kb region of C1orf64 and SPDEF promoters was carried out using PROMO software, which employs TRANSFAC version 8.3 [53, 54]. The sequence of each 2 kb promoter was obtained using Ensembl Genome Browser (http://www.ensembl.org/index.html). Binding sites were predicted within a dissimilarity margin of ≤15%. Random Expectation (RE) gives the number of expected occurrences of the match in a random sequence of the same length as the query sequence according to the dissimilarity index. Two models are considered: equiprobability for the 4 nucleotides (RE equally) and estimating the nucleotide probability as the nucleotide frequencies in the query sequence (RE query).

#### Analysis for open chromatin regions

Data for DNase I Hypersensitivity Sites (DHSs) are from ENCODE project that includes a uniform set of open chromatin elements in 125 cell types based on DNase-seq data [55, 56]. Data for H3K27ac Mark are generated at the Broad Institute and Bernstein lab at Massachusetts General Hospital/Harvard Medical School [57, 58]. Clustering of results and Track Data Hubs visualization were carried out at the University of California Santa Cruz (UCSC), [59–62]. Data analysis for DHS and H3K27ac Mark on C1orf64 and SPDEF promoters were performed using the UCSC Genome Browser (http://genome.ucsc.edu/) and (http://www.epigenomebrowser.org/).

#### Analysis of ChIP-seq data

AR ChIP-seq data for AR-positive breast cancer cell line MDA-MB-453 was accessed using ChIP-Atlas browser (http://chip-atlas.org/). Three replicate experiments for AR ChIP-seq were extracted from a study by Malinen *et al*. [63]. The data from these replicates were analyzed to identify the subset of AR-signature genes that have a detectable AR-binding site within their 5 kb promoter regions. For the signature genes with a detectable AR-binding, an average of ChIP-seq signal was obtained across the replicates. Next, Pearson CC was calculated between C1orf64 expression and each gene in this identified subset of AR-signature. Datasets from breast cancer cell lines were applied to obtain CC values and for genes that were not present on the same platform as C1orf64, CC values were calculated from TCGA and Bos *et al*. datasets using *ONCOMINE* (www.oncomine.org), [21–25].

#### Protein motif analysis

Scansite software was employed to identify motifs within C1orf64 protein that are likely to be phosphorylated by specific protein kinases or bind to domains such as SH2 domains, 14-3-3 domains or PDZ domains (http://scansite.mit.edu/), [64, 65]. C1orf64 protein sequence was obtained from Ensembl genome browser and motif scan was carried out with high stringency (best 0.2% of all sites) using C1orf64 sequence. Next, using *Scansite* analysis, site of each motif, sequence score and percentile for this score (percentile of score when compared to all records used in this search), sequence of each motif, and surface accessibility of the predicated site were obtained.

### Cell culture

Breast cancer cell lines T-47D (ER-positive/AR-positive) and MFM-223 (ER-negative/AR-positive) were obtained from (Sigma-Aldrich, St. Louis, MO, USA), [14, 20]. Culture media were obtained from Life Technologies (Grand Island, NY, USA). T-47D and MFM-223 cell lines were cultured in DMEM/F12 and DMEM media, respectively supplemented with 10% fetal bovine serum (FBS), (Fisher Scientific, Waltham, MA, USA). Cell culture treatment with 5α-dihydrotestosterone (Sigma-Aldrich) was carried out at 10 nM concentration at 6h, 24h, and 48h time-points for gene expression studies, which is the standard DHT concentration used for AR activation in breast cancer cell lines [10, 66, 67]. The stated time-points for DHT treatment have been previously applied for the study of AR-response genes [8, 10, 20, 68]. Treatment of T-47D cells with estradiol (E2, Sigma-Aldrich) was performed at 10 nM concentration for 24h. DHT and E2 treatments were performed in phenol red-free media (Life Technologies) supplemented with 10% Charcoal/Dextran treated serum (Fisher Scientific) and cell lines were cultured in the media 48h prior to DHT and E2 treatments [8, 20]. AR inhibition with flutamide (Sigma-Aldrich) was carried out at 10 μM concentration for 24h in medium containing 10% FBS as described before [9]. AR inhibition with enzalutamide was performed at 10 μM concentration for 24h in full media as suggested by the supplier (Selleck Chemicals, Houston, TX, USA). Control experiments were carried out using an equal volume of solvent for each compound.

### Quantitative real time-polymerase chain reaction

The gene expression levels were assessed using qRT-PCR with the following Taqman Gene Expression Assays (Life Technologies) as instructed by the manufacturer: C1orf64 (assay ID: Hs00698851_m1), SIDT1 (assay ID: Hs00214475_m1), RHOH (assay ID: Hs01877256_s1), TFAP2B (assay ID: Hs01560931_m1), SPDEF (assay ID: Hs01026050_m1), PIP (assay ID: Hs01114172_m1), and PGR (assay ID: Hs01556702_m1). Housekeeping gene RPLP0 (Life Technologies) was applied as control. Fold change in gene expression = gene expression in treated group/ average gene expression in control group [8, 14, 20]. Down-regulation of genes are presented in the text as a fold reduction relative to controls.

### Western blot analysis

Rabbit polyclonal C1orf64 antibody (Novus Biologicals, Littleton, CO, USA) and rabbit polyclonal SPDEF antibody (Life Technologies) were applied at 1:250 dilutions. Rabbit polyclonal AR antibody (Active Motif, Carlsbad, CA, USA) and rabbit polyclonal 14-3-3 (pan) antibody (Millipore, Temecula, CA, USA) were used at 1:1000 and 1:5000 dilutions, respectively. Rabbit monoclonal PIP antibody (Novus Biologicals) was applied at 1:1000 dilution. Mouse monoclonal α-tubulin antibody (Sigma-Aldrich) was applied at 1:2000 dilution to assess loading. Protein concentrations were measured using the BCA Protein Assay Kit (Thermo Fisher Scientific, Waltham, MA, USA) and a total of 20 μg of each cell lysate was used for immunoblotting. Immunoblot imaging and analysis of band densities were performed by a C-DiGit Blot Scanner (LI-COR, Lincoln, NE, USA). Western blots were performed in three replicates and the average fold changes were shown.

### Chromatin immunoprecipitation assay

ChIP assays were carried out in T-47D and MFM-223 cell lines using QuickChIP kit (Novus Biologicals) as instructed by the manufacturer [20]. Cell lines were treated with 10 nM of DHT for 24h before ChIP assays in phenol red-free media supplemented with 10% Charcoal/Dextran treated serum. DNA shearing was carried out by sonication at 50% output with fifteen pulses of 10 seconds each and a 1 minute rest on ice between each pulse using a Model 120 Sonic Dismembrator (Fisher Scientific). A total of 5 μg of ChIP-grade rabbit polyclonal AR antibody (Active Motif) was utilized for each ChIP assay. To quantify ChIP results, primer sets for each promoter were applied for qRT-PCR amplification by SYBR green method (Applied Biosystems) as published before [20, 69]. The sequence of the 2 kb promoter region for each gene was obtained using Ensembl genome browser and primer sets were designed using Primer3web version 4.0.0 (http://bioinfo.ut.ee/primer3/), [70]. In this process, six primer sets with the most optimal characteristics were selected to cover each 2 kb promoter region of C1orf64 and SPDEF for ChIP assays.

For C1orf64 promoter the following primer sets were applied: Amplicon 1 (A1), (-112 to -11 bp): forward (F) primer 1, atcaaagcgatcccctgact and reverse (R) primer 1, tgcgggaccagaccttttat; A2 (-496 to -410 bp): F2, ttcaccgtgttagccaggat and R2, catgcctgtaatcccagcac; A3 (-828 to -762 bp): F3, ggaggtggaggtttcagtga and R3, gtttcgctcttcttgcccag; A4 (-1019 to -890 bp): F4, tcacacctgtaatcccagca and R4, ccacacccggctaattttgt; A5 (-1648 to -1527 bp): F5, : agctcacccacgtaatccag and R5, ccacgccttaatccccaaag; A6 (-1896 to -1840 bp): F6, tcccaggttcaagcgattct and R6, cgcctgtaatcccagctact. For SPDEF promoter the following primer sets were used: A1 (-167 to -82 bp): F1, ctagctgtcagggcatggat and R1, tgcttacctcagaccacagg; A2 (-400 to -315 bp): F2, atttccctagttggtgccca and R2, aagtgagcagcagtggagat; A3 (-955 to -847 bp): F3, ccccagaatcctcatgctct and R3, cacgtacctgctacctccat; A4 (-1196 to -1063 bp): F4, ggggattaggagtggtcgag and R4, tgttatcgtcactggcacct; A5 (-1704 to -1626 bp): F5, tgggttcaagcgattctcct and R5, aaaattagctgggcgtggtg; A6 (-1957 to -1895 bp): F6, gaggccgaggtacaaggatt and R6, tctctatgttgcccagggtg. For PIP promoter the following primer sets were employed: A1 (-242 to -95 bp): F1, aggtcccagccattttgaga and R1, ccccactcgtgatctttcct; A2 (-954 to -905 bp): F2, acaatgccagtgtcagcaag and R2, cctctccttgggatgatggg; A3 (-1533 to -1442 bp): F3, atgccttcaaagagccaagc and R3, actgaccaaggcaggcataa; A4 (-1960 to -1876 bp): F4, tcacacctgtaatcccagca and R4, catgttggccaagctgatct.

Amplification of 1% of input chromatin prior to immunoprecipitation was applied as the input control and ChIP assays using a non-specific antibody (rabbit IgG) served as a negative control [20]. Fold enrichments for ChIP-signal were calculated as described before [20, 71]. First the normalized ChIP *C*_t_ values were calculated: Δ*C*_t (normalized ChIP)_ = *C*_t (ChIP)_ – [*C*_t (Input)_ – Log_2 (Input Dilution Factor)_]. Next, the % Input was calculated as: % Input = 2 ^(-ΔCt [normalized ChIP])^. Lastly, fold enrichment was calculated: % Input of antibody/ % Input of IgG [71].

### RNA interference

C1orf64-silencing by RNA interference in T-47D and MFM-223 cell lines was performed by the reverse transfection method using Lipofectamine RNAiMAX (Life Technologies), [14, 72]. Two sets of siRNA-duplexes (Sigma-Aldrich) were applied for C1orf64-silencing: siRNA-D1: sense (s), GCGCCUGUGAGGUCUUCAA[dT][dT]; anti-s, UUGAAGACCUCACAGGCGC[dT][dT]; siRNA-D2: s, GCCUGUGAGGUCUUCAACU[dT][dT]; anti-s, AGUUGAAGACCUCACAGGC[dT][dT]. AR-silencing in cell lines was carried out using an AR-siRNA duplex (Sigma-Aldrich): s, CCAUCUUUCUGAAUGUCCU[dT][dT]; anti-s, AGGACAUUCAGAAAGAUGG[dT][dT]. Transfections of siRNA Universal Negative Control # 1 (Sigma-Aldrich) were used as control. The effect of siRNA-silencing was assessed seventy-two hours after transfections.

### C1orf64 overexpression

C1orf64 open reading frame (ORF) clone in a pReciever-M02 plasmid was obtained from GeneCopoeia (Rockville, MD, USA). Transfection of C1orf64 expression construct in T-47D and MFM-223 cell lines was carried out using TurboFect Transfection Reagent (Thermo Fisher Scientific) as instructed by the manufacturer. An empty pReciever-M02 plasmid was applied for the control experiments. The effect of C1orf64 overexpression was assessed 48h following transfections.

### AR reporter assays

T-47D and MFM-223 cell lines were cultured in solid opaque (white) 96-well tissue culture plates (Fisher Scientific) using phenol red-free media (Life Technologies) supplemented with 10% Charcoal/Dextran treated serum (Fisher Scientific) and cell lines were approximately 80% confluent on the day of transfection. AR Reporter Assay Kit (Cignal, Cat. 336841) was obtained from Qiagen (Valencia, CA, USA). AR reporter includes a mixture of an inducible androgen-responsive reporter construct and a constitutively expressing Renilla construct (40:1). This reporter encodes the firefly luciferase reporter gene under the control of a basal promoter element (TATA box) joined to tandem repeats of an Androgen Response Element (ARE). Negative control is a mixture of a non-inducible firefly reporter construct and a constitutively expressing Renilla construct (40:1).

The co-transfections of reporter and expression constructs in each cell line were carried out using TurboFect Transfection Reagent (Thermo Fisher Scientific) as instructed by the manufacturer in the following four experimental groups: 1) Negative control for reporter + empty pReciever-M02 plasmid (Neg-Rep/Vec), 2) Negative control for reporter + C1orf64 expression construct (Neg-Rep/C1orf64), 3) AR reporter + empty pReciever-M02 plasmid (Pos-Rep/Vec), and 4) AR reporter + C1orf64 expression construct (Pos-Rep/C1orf64). Twenty-four hours following co-transfections, media were changed to phenol red-free media/10% Charcoal/Dextran treated serum containing DHT at 10 nM concentration. Forty-eight hours after the transfections, reporter activities were assessed using Dual-Glo Luciferase Assay System (Promega, Madison, WI, USA) as instructed by the manufacturer and measurements were carried out in an EnVision Multilabel Reader (Perkin Elmer, Waltham, MA, USA). The relative response ratio (RRR) for each reporter activity was measured relative to the internal control reporter followed by normalization to the respective Neg-Rep group [8, 9]. All reporter assays were carried out in four biologic replicates.

### Co-immunoprecipitation assay

IP assays for endogenous AR and 14-3-3 were carried out using a non-denaturing lysis buffer as we previously published [8, 14]. The IP lysis buffer constituted of 20 mM Tris Hcl pH 8, 137 nM NaCl, 10% glycerol, 1% Nonidet P-40 (NP-40), and 2mM EDTA [14]. For IP experiments, T-47D and MFM-223 cell lines were grown to 70% confluence in 10 cm dishes in full media and then each dish was lysed in 1 ml of IP lysis buffer supplemented with protease and phosphatase inhibitors (Sigma Aldrich). Following rotatory agitation of samples for 30 min at 4°C, lysates were centrifuged at 14,000 xg for 20 min and supernatants were collected. Next, lysates were each pre-cleared with 100 μl of Protein A-Sepharose 4B beads (Life Technologies) for 30 min at 4°C followed by centrifugation and collection of supernatants for IP experiments.

Subsequently, AR-IP was performed using 10 μg of rabbit polyclonal AR antibody (Active Motif) and 14-3-3 IP was carried out using 5 μg of rabbit polyclonal 14-3-3 (pan) antibody (Millipore). Control experiments were conducted with a non-specific rabbit IgG. The lysate-antibody mixtures were then incubated overnight under rotation at 4°C. Next, lysates were mixed with 100 μl of beads for 4h under rotatory agitation at 4°C and then centrifuged to remove supernatants. Beads were then washed three times with ice-cold IP lysis buffer. Following the removal of last supernatant, 50 μl of 2x Laemmli Sample Buffer (Bio-Rad, Hercules, CA, USA) was added to each sample. Samples were then boiled at 100°C for 5 min and centrifuged at 14,000 xg for 10 min. Finally, supernatants were collected and applied for immunoblot analysis using C1orf64, AR, or 14-3-3 antibodies. In addition, for each sample, 5% of lysate was collected before IP to assess input by immunoblot analysis. All co-IP experiments were carried out in three replicates.

### Statistical analysis

Biostatistics was carried out using IBM SPSS Statistics 23. Unless specified, Student t-test and paired sample t-test were applied to calculate the statistical significance. For the analysis of association between C1orf64 expression and clinical and pathological features, p values were calculated using Wilcoxon Signed Rank Test. All error bars depict ± 2SEM.

## SUPPLEMENTARY MATERIALS FIGURES AND TABLES





## Abbreviations

AR: androgen receptor
ER: estrogen receptor
PIP: Prolactin-Induced Protein
PGR: progesterone receptor
ARE: androgen response element
DHT: dihydrotestosterone
E2: estradiol
ERK: extracellular signal-regulated kinase
qRT-PCR: quantitative real time-polymerase chain reaction
ChIP: chromatin immunoprecipitation
IP: immunoprecipitation
DAVID: Database for Annotation, Visualization and Integrated Discovery
GO: Gene Ontology
CC: Pearson correlation coefficient
siRNA-D1: C1orf64 siRNA-duplex1
siRNA-D2: C1orf64 siRNA-duplex2
ChIP-seq: ChIP-sequencing
