# Supplementary material for: C1orf64 is a novel androgen receptor target gene and coregulator that interacts with 14-3-3 protein in breast cancer
Source: Oncotarget. 2017 May 11;8(34):57907–33. doi: 10.18632/oncotarget.17826 (PMC5593696; doi:10.18632/oncotarget.17826)
Supplement: Supplementary file 1 [file oncotarget-08-57907-s001.pdf]

C1orf64 is a novel androgen receptor target gene and coregulator that interacts with 14-3-3 protein in breast cancer

SUPPLEMENTARY FIGURES AND TABLE

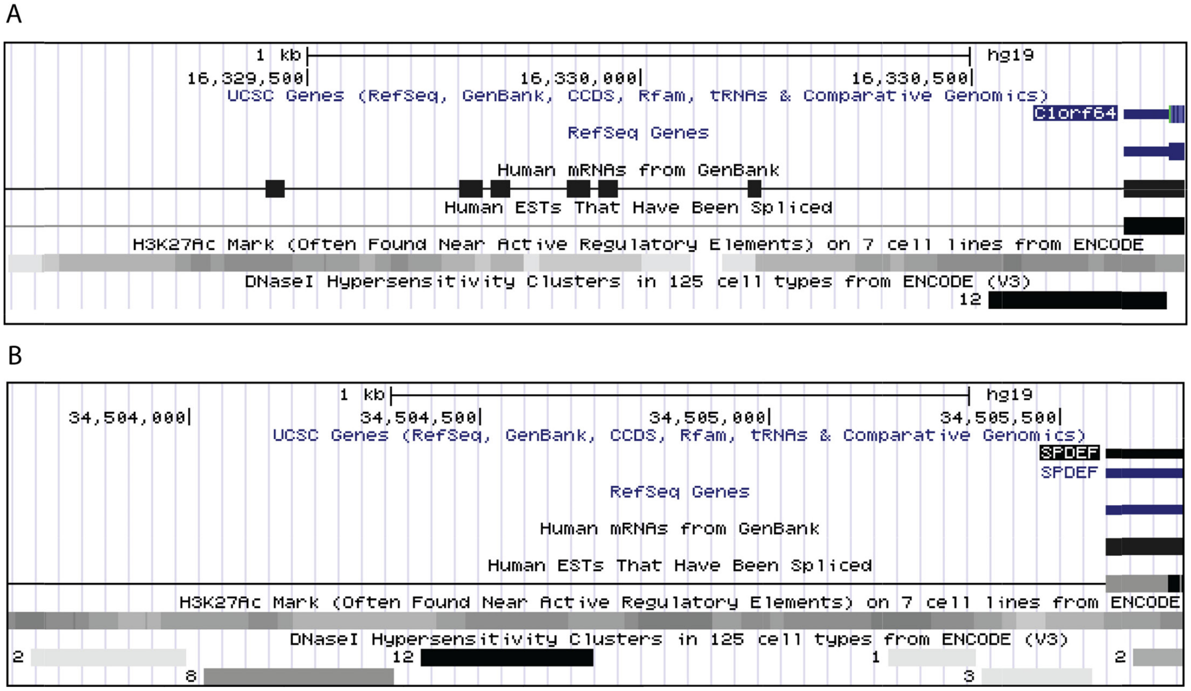

Supplementary Figure 1: DNase I hypersensitive sites (DHSs) and H3K27ac Mark on C1orf64 and SPDEF promoters. **(A)** Demonstrates DHSs clusters and H3K27ac Mark locations on the 1.8 kb region of C1orf64 promoter obtained from data of ENCODE project using the UCSC Genome Browser (http://genome.ucsc.edu/) and (http://www.epigenomebrowser.org/). For each DHS cluster a gray box indicates the extent of the hypersensitive region. The darkness is proportional to the maximum signal strength observed in any cell line. The number to the left of the box shows how many cell lines are hypersensitive in the region. H3K27ac track shows the levels of enrichment of the H3K27Ac histone mark across the genome as determined by a ChIP-seq assay. This track uses a transparent overlay method of displaying data from cell lines in the same vertical space. **(B)** Demonstrates DHSs clusters and H3K27ac Mark locations on the 1.8 kb region of SPDEF promoter the UCSC Genome Browser as explained in **(A)**.

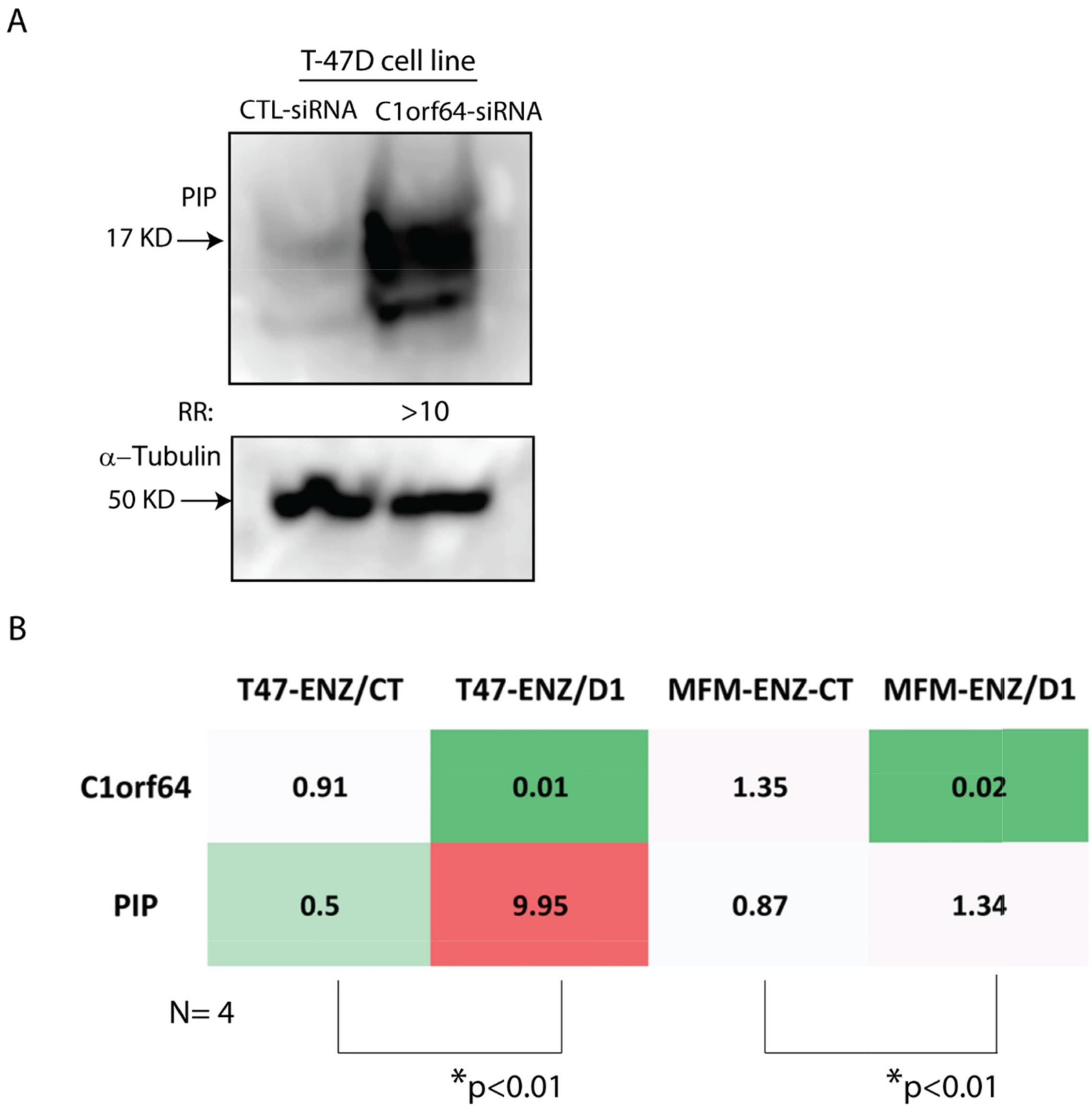

Supplementary Figure 2: PIP protein level following C1orf64 silencing and the effect of combined enzalutamide treatment and C1orf64 silencing on PIP expression. (A) immunoblotting to assess PIP protein level following C1orf64 silencing with siRNA-D1 (C1orf64-siRNA) in T-47D cell line. Fold change (RR) band density was measured relative to control-siRNA (CTL-siRNA) in three replicate experiments. A mouse α-tubulin antibody was applied to assess loading. **(B)** A Heat map to show fold changes in C1orf64 and PIP expression using qRT-PCR after enzalutamide (ENZ) treatment at 10 µM and C1orf64 silencing with siRNA-D1 (D1) in T-47D and MFM-223 cell lines. Fold changes were assessed relative to control-siRNA/solvent-treated group. CT: control-siRNA. *, $p < 0.01$ is for ENZ/D1 vs. ENZ/CT groups. Red color denotes up-regulation and green color indicates down-regulation.

Supplementary Table 1A: A list of highly co-expressed genes with AR in study-1

Gene	CC (dataset-1)	p value	CC (dataset-2)	p value
AR	1	p< 0.001	1	p< 0.001
F7	0.716	p< 0.001	0.521	p< 0.001
PATZ1	0.709	p< 0.001		
ZNF205-AS1	0.699	p< 0.001		
NFATC4	0.681	p< 0.001	0.253	p=0.08
SPDEF	0.67	p< 0.001	0.655	p< 0.001
MXD4	0.661	p< 0.001		
CTNND2	0.651	p< 0.001	0.566	p< 0.001
SLCO2A1	0.645	p< 0.001	0.08	p> 0.1
RHOH	0.643	p< 0.001	0.693	p< 0.001
SGSM3	0.638	p< 0.001		
TP53TG1	0.636	p< 0.001		
FGFR4	0.634	p< 0.001	0.541	p< 0.001
PYGO1	0.633	p< 0.001	-0.379	p= 0.02
MGAT5	0.632	p< 0.001	0.06	p> 0.1
PCDHA5	0.621	p< 0.001		
DALRD3	0.616	p< 0.001		
SLC9A1	0.614	p< 0.001		
IGHM	0.612	p< 0.001	-0.02	p> 0.1
AMBP	0.603	p< 0.001	0.25	p= 0.1
CRAT	0.601	p< 0.001	0.39	p= 0.007
MVK	0.601	p< 0.001		
BUB1	-0.609	p< 0.001	-0.301	p= 0.04
GART	-0.609	p< 0.001		
STIL	-0.612	p< 0.001		
TOP2A	-0.612	p< 0.001	-0.238	p= 0.1
GLS	-0.613	p< 0.001	-0.442	p= 0.002
PGM1	-0.628	p< 0.001	-0.484	p= 0.001
GALNT2	-0.63	p< 0.001	-0.495	p< 0.001
USP1	-0.642	p< 0.001		
NAB1	-0.644	p< 0.001	-0.671	p< 0.001
PICALM	-0.649	p< 0.001		
EHBP1	-0.654	p< 0.001	-0.493	p< 0.001
TTK	-0.662	p< 0.001	-0.287	p= 0.05
DONSON	-0.681	p< 0.001		
PRNP	-0.75	p< 0.001	-0.632	p< 0.001

All listed genes have an absolute correlation coefficient lCCl ≥ 0.6 with AR expression at a p< 0.001 in study-1. The corresponding correlation coefficient and p value for each gene in the list, which is also present in study-2, are shown.

Supplementary Table 1B: A list of highly co-expressed genes with AR in study-2. All listed genes have an absolute correlation coefficient lCCl ≥ 0.6 with AR expression at a $p < 0.001$ in study-2. The corresponding correlation coefficient and p value for each gene in the list, which is also present in study-1, are shown.

See Supplementary File 1

Supplementary Table 1C: A combined list of highly co-expressed genes with AR in two datasets. All listed genes have an absolute correlation coefficient lCCl values ≥ 0.6 with AR expression at a $p < 0.001$ in at least one dataset. Asterisks indicate genes that are present in both datasets and have a significant CC ($p < 0.05$) with AR. The strongest CC values for these genes are shown.

See Supplementary File 1
