# Supplementary material for: C1orf64 is a novel androgen receptor target gene and coregulator that interacts with 14-3-3 protein in breast cancer
Source: Oncotarget. 2017 May 11;8(34):57907–33. doi: 10.18632/oncotarget.17826 (PMC5593696; doi:10.18632/oncotarget.17826)
Supplement: Supplementary file 2 [file oncotarget-08-57907-s002.pdf]

Supplementary Table 1 (B). A list of highly co-expressed genes with AR in study-2. All listed genes have an absolute correlation coefficient lCCl ≥ 0.6 with AR expression at a p< 0.001 in study-2. The corresponding correlation coefficient and p value for each gene in the list, which is also present in study-1, are shown.

Gene	CC (dataset-2)	p value	CC (dataset-1)	p value
AR	1	p< 0.001	1	p< 0.001
C1orf64	0.737	p< 0.001		
SIDT1	0.73	p< 0.001		
RHOH	0.693	p< 0.001	0.643	p< 0.001
CCDC125	0.689	p< 0.001		
LRFN2	0.678	p< 0.001		
TBC1D30	0.673	p< 0.001		
TFAP2B	0.673	p< 0.001	0.484	p< 0.001
BCAS1	0.662	p< 0.001	0.462	p< 0.001
IRX5	0.659	p< 0.001	0.514	p< 0.001
CACNA1D	0.656	p< 0.001	0.477	p< 0.001
DOPEY2	0.656	p< 0.001		
SPDEF	0.655	p< 0.001	0.67	p< 0.001
RHOB	0.654	p< 0.001	0.587	p< 0.001
FOXA1	0.644	p< 0.001	0.574	p< 0.001
FOXR1	0.644	p< 0.001		
SLC9A2	0.637	p< 0.001		
TTC6	0.631	p< 0.001		
EMP2	0.63	p< 0.001	0.375	p= 0.006
CREB3L4	0.629	p< 0.001		
SLC16A6	0.628	p< 0.001	-0.231	p= 0.01
KIAA1324	0.627	p< 0.001	0.339	p= 0.01
GGCT	0.623	p< 0.001		
DEGS2	0.622	p< 0.001		
TRIL	0.62	p< 0.001		
REEP6	0.615	p< 0.001		
UMODL1	0.614	p< 0.001		
HPX	0.612	p< 0.001	0.493	p< 0.001
GATA3	0.61	p< 0.001	0.553	p< 0.001
PLEKHF2	0.61	p< 0.001		
TFF3	0.607	p< 0.001	0.401	p= 0.003
GSE1	0.605	p< 0.001		
RND1	0.605	p< 0.001		
ZG16B	0.603	p< 0.001		
TPRN	0.602	p< 0.001		
CACFD1	0.601	p< 0.001	0.485	p< 0.001
SLC9A3R1	0.601	p< 0.001	0.49	p< 0.001
ATP8A1	0.6	p< 0.001	0.169	p> 0.1
HMGCS2	0.6	p< 0.001	0.5	p< 0.001
CMTM7	-0.603	p< 0.001		
NXN	-0.604	p< 0.001		
ANXA1	-0.609	p< 0.001	-0.425	p= 0.002
CENTD3	-0.609	p< 0.001		
WIPF1	-0.609	p< 0.001		
LINC00597	-0.612	p< 0.001	-0.332	p= 0.016

AKR1B1	-0.614	p< 0.001	-0.466	p< 0.001
PIM1	-0.615	p< 0.001	-0.433	p= 0.001
FHL3	-0.617	p< 0.001	-0.101	p> 0.1
CAPG	-0.622	p< 0.001	-0.192	p> 0.1
CAV1	-0.622	p< 0.001	-0.375	p= 0.006
POPDC3	-0.624	p< 0.001		
PRNP	-0.632	p< 0.001	-0.75	p< 0.001
FYN	-0.633	p< 0.001	-0.375	p= 0.006
TUBB6	-0.634	p< 0.001		
CAV2	-0.637	p< 0.001	-0.516	p< 0.001
ncRNA miR-221	-0.639	p< 0.001		
ANKS6	-0.641	p< 0.001		
INPP1	-0.652	p< 0.001	-0.428	p= 0.002
S100A3	-0.655	p< 0.001	-0.337	p= 0.015
KIRREL	-0.661	p< 0.001	0.105	p> 0.1
ECM2	-0.663	p< 0.001	0.106	p> 0.1
IGF2BP2	-0.664	p< 0.001		
NAB1	-0.671	p< 0.001	-0.644	p< 0.001
ETS1	-0.672	p< 0.001	-0.045	p> 0.1
APOBEC3C	-0.673	p< 0.001		
NFIL3	-0.674	p< 0.001	-0.403	p= 0.003
LARP6	-0.679	p< 0.001		
LAMA2	-0.686	p< 0.001	-0.155	p> 0.1

Supplementary Table 1 (C). A combined list of highly co-expressed genes with AR in two datasets. All listed genes have an absolute correlation coefficient lCCl values ≥ 0.6 with AR expression at a $p < 0.001$ in at least one dataset. Asterisks indicate genes that are present in both datasets and have a significant CC ($p < 0.05$) with AR. The strongest CC values for these genes are shown.

Gene (combined)	Correlation Coefficient (CC)
AR	1
C1orf64	0.737
SIDT1	0.73
*F7	0.716
PATZ1	0.709
ZNF205-AS1	0.699
*RHOH	0.693
CCDC125	0.689
NFATC4	0.681
LRFN2	0.678
TBC1D30	0.673
*TFAP2B	0.673
*SPDEF	0.67
*BCAS1	0.662
MXD4	0.661
*IRX5	0.659
*CACNA1D	0.656
DOPEY2	0.656
*RHOB	0.654
*CTNND2	0.651
SLCO2A1	0.645
*FOXA1	0.644
FOXR1	0.644
SGSM3	0.638
SLC9A2	0.637
TP53TG1	0.636
*FGFR4	0.634
PYGO1	0.633
MGAT5	0.632
TTC6	0.631
*EMP2	0.63
CREB3L4	0.629
*SLC16A6	0.628
*KIAA1324	0.627
GGCT	0.623
DEGS2	0.622
PCDHA5	0.621
TRIL	0.62
DALRD3	0.616
REEP6	0.615
SLC9A1	0.614
UMODL1	0.614
IGHM	0.612

*HPX	0.612
*GATA3	0.61
PLEKHF2	0.61
*TFF3	0.607
GSE1	0.605
RND1	0.605
AMBP	0.603
ZG16B	0.603
TPRN	0.602
*CRAT	0.601
MVK	0.601
*CACFD1	0.601
*SLC9A3R1	0.601
ATP8A1	0.6
*HMGCS2	0.6
CMTM7	-0.603
NXN	-0.604
*BUB1	-0.609
GART	-0.609
*ANXA1	-0.609
CENTD3	-0.609
WIPF1	-0.609
STIL	-0.612
TOP2A	-0.612
*LINC00597	-0.612
*GLS	-0.613
*AKR1B1	-0.614
*PIM1	-0.615
FHL3	-0.617
CAPG	-0.622
*CAV1	-0.622
POPDC3	-0.624
*PGM1	-0.628
*GALNT2	-0.63
*FYN	-0.633
TUBB6	-0.634
*CAV2	-0.637
ncRNA miR-221	-0.639
ANKS6	-0.641
USP1	-0.642
PICALM	-0.649
*INPP1	-0.652
*EHBP1	-0.654
*S100A3	-0.655
KIRREL	-0.661
TTK	-0.662
ECM2	-0.663
IGF2BP2	-0.664
*NAB1	-0.671

ETS1	-0.672
APOBEC3C	-0.673
*NFIL3	-0.674
LARP6	-0.679
DONSON	-0.681
LAMA2	-0.686
*PRNP	-0.75
